# Molecular scale nanophotonics: hot carriers, strong coupling, and electrically driven plasmonic processes

**DOI:** 10.1515/nanoph-2023-0710

**Published:** 2024-03-28

**Authors:** Yunxuan Zhu, Markus B. Raschke, Douglas Natelson, Longji Cui

**Affiliations:** Department of Physics and Astronomy, 3990 Rice University, Houston, TX, USA; Department of Physics, and JILA, 1877 University of Colorado Boulder, Boulder, CO, USA; Department of Physics and Astronomy, Electrical and Computer Engineering, Materials Science and Nanoengineering, 3990 Rice University, Houston, TX, USA; Department of Mechanical Engineering, Materials Science and Engineering Program, & Center for Experiments on Quantum Materials (CEQM), 1877 University of Colorado Boulder, Boulder, CO, USA

**Keywords:** plasmonics, hot carriers, strong coupling, molecular nanophotonics, quantum tunnelling junctions

## Abstract

Plasmonic modes confined to metallic nanostructures at the atomic and molecular scale push the boundaries of light–matter interactions. Within these extreme plasmonic structures of ultrathin nanogaps, coupled nanoparticles, and tunnelling junctions, new physical phenomena arise when plasmon resonances couple to electronic, exitonic, or vibrational excitations, as well as the efficient generation of non-radiative hot carriers. This review surveys the latest experimental and theoretical advances in the regime of extreme nano-plasmonics, with an emphasis on plasmon-induced hot carriers, strong coupling effects, and electrically driven processes at the molecular scale. We will also highlight related nanophotonic and optoelectronic applications including plasmon-enhanced molecular light sources, photocatalysis, photodetection, and strong coupling with low dimensional materials.

## 1 Introduction

**Figure 1:**
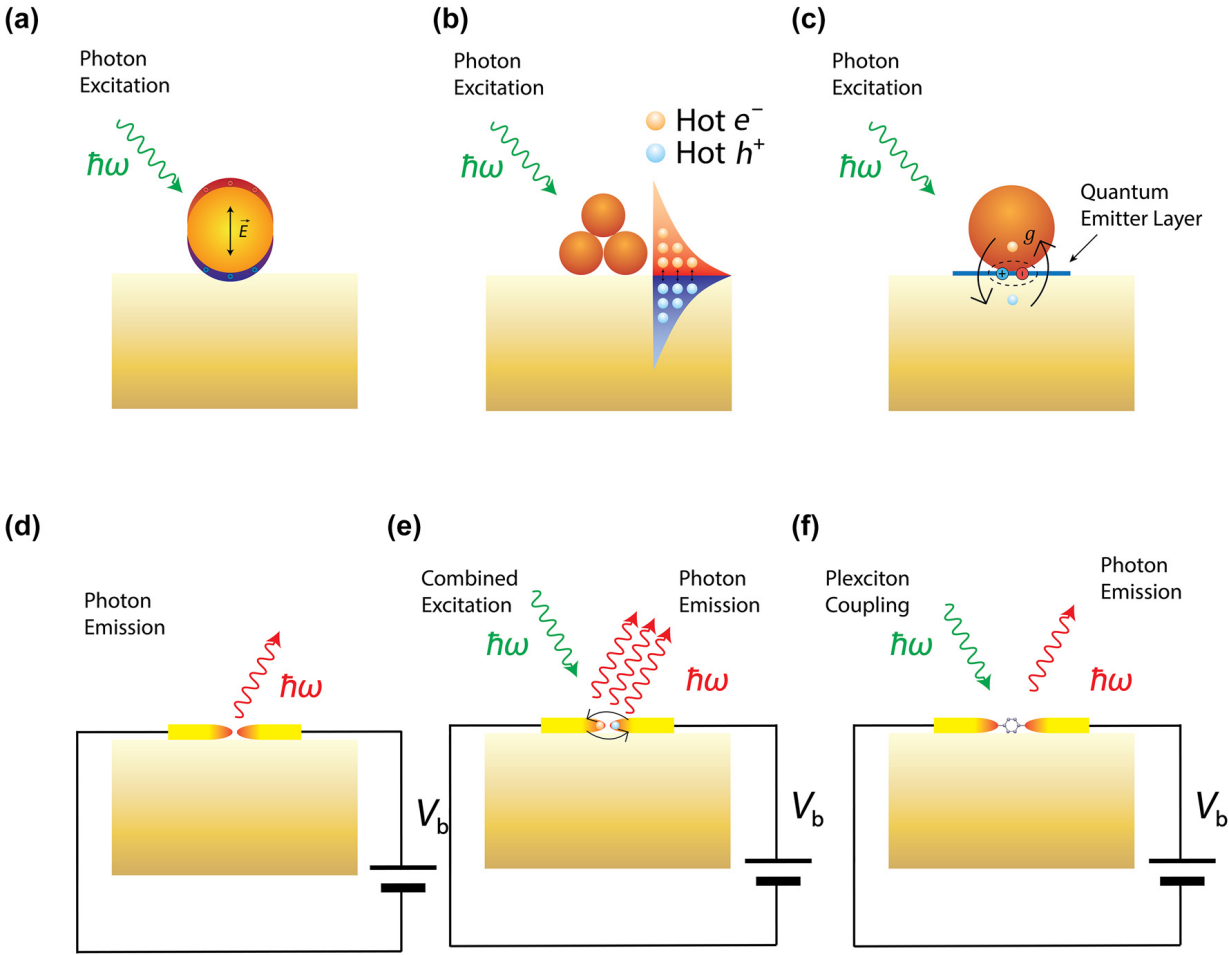
Optically or electrically driven plasmonic nanostructures. (a) Photon excitation induced electrical field enhancement inside a nanoparticle (Purcell effect). (b) Photon excitation induced hot carrier generation in plasmonic nano structures. (c) Plasmon-exciton coupling probed through optical excitation. (d) Electrically driven plasmonic tunnel junction for light emission. (e) Combined electrical and optical excitation for enhanced hot carrier induced light emission. (f) Combined excitation for probing plexciton strong coupling in molecular scale plasmonic systems.

Localized surface plasmon resonances (LSPR) are the surface confined collective and coherent oscillations of conduction electrons of metallic nanostructures, often excited when illuminated by light (Figure 1(a)). This phenomenon results in the strong spatial confinement of electromagnetic fields to dimensions much smaller than the free-space wavelength of light [1], [2], [3], [4]. LSPRs are characterized by several remarkable physical properties, such as enhanced electric fields, optical phase control, strong resonant absorption and scattering, and high sensitivity to changes in the local dielectric environment – or fundamentally a modification of the local electromagnetic density of states analogous to cavity optics arising from Purcell enhancement to strong coupling. By manipulating size, shape, and material composition of the nanostructure, the LSPR can be tuned across a wide spectral range from the ultraviolet to the infrared. Since the pioneering work of Rufus Ritchie in the 1950s predicting surface plasmons [5], LSPR research has proliferated. Progress has largely been driven by synthesis and fabrication of metallic nanostructures, near-field scanning optical microscopy techniques, and precise theory and predictive computational modelling [6], [7]. A wide range of engineered plasmonic nanostructures made of different types of metals and a variety of shapes have paved the way for an exceptionally diverse range of applications such as surface-enhanced spectroscopy, deep-subwavelength spatial resolution imaging in tip-enhanced Raman scattering (TERS) and a wide range of other linear and nonlinear optical processes [8], [9], [10], [11], [12], [13], [14], biological and chemical molecular sensing [15], light harvesting [16], and medical diagnostics and therapy [17].

Since LSPR can be excited by optical illumination, inelastic electron tunneling, or both, the associated hot carrier dynamics correspondingly can be driven optically, electrically, or via their hybrid interaction. For electrically driven hot carrier processes in particular, the generation of LSPRs often occurs in ultranarrow atomic scale plasmonic nanogaps or nanojunctions [18]. These structures can be treated as two coupled individual plasmonic nanostructures bridged by a quantum tunneling junction. Such configurations have recently been studied in molecular scale nanophotonics, in which the optical fields can be ultra-confined down to near-atomic scale dimensions (with volumes below 1 nm^3^), featuring Purcell enhancement (Figure 1(d)), giant photon upconversion (Figure 1(e)) [19], and even strong coupling (Figure 1(f)) [20], [21]. In contrast, simple individual plasmonic nanostructures like nanoparticles and nanowires can only confine light to comparatively large volumes determined by the minimum feature sizes of typically few 10s of nm. The fact that such atomic scale gap-coupled plasmonic processes are naturally compatible with molecular dimensions lends the great opportunities for previously inaccessible regimes such as strong plexcitonic coupling and quantum plasmonics [22], [23], [24], [25].

Recently, other intriguing aspects of LSPR have garnered great interest: their ability to produce nonequilibrium energetic “hot” carriers including hot electrons and hot holes on the nanoscale [26]. In general, and as well-established from ultrafast spectroscopy of metal surfaces, the ultrafast generation of LSPRs is followed by both their radiative and non-radiative relaxation decay, leading to the radiative emission of photons and non-radiative generation of hot carriers (Figure 1(b)), transiently and on femtosecond timescales. Interestingly, the LSPR mediated generation of hot carriers has distinct attributes and is more efficient than the direct photon absorption in metals as a result of the substantial absorption cross-section associated with plasmonic nanostructures [27], which can surpass the geometrical cross section by several orders of magnitude and could potentially facilitate strong light matter interactions (Figure 1(c)) [23], [28]. These hot carriers induced by LSPR are of interest for potentially new pathways in photochemistry [29], [30], [31], photodetection [32], [33], photoluminescence [34], and photovoltaics [1], [35].

In this review, we will focus on recent advances in plasmon-induced hot carriers and electrically driven plasmonic processes at the atomic and molecular scale. We will highlight our current theoretical understanding of hot carrier generation and relaxation dynamics. Recent developments of advanced experimental tools enabling hot carrier detection and imaging will be described. We will also discuss several important applications such as molecular scale photon emission, photocatalysis, opto-electronic sensing, and strong coupling-enabled molecular nano-photonics. While optically driven plasmonics and hot carrier processes have been studied extensively in recent years on nanoscale dimensions, their ultimate nanoscale extension (“picocavities”) and in particular electrically driven counterparts have received comparatively less attention. Notably, an electrically driven approach offers several benefits including broadband hot carrier excitation, the ability to excite conventionally optical dipole excitation-forbidden dark plasmons, and seamless integration with on-chip optoelectronics. This complements attributes of, e.g., femtosecond and near-field optical excitation. Unique to electrically driven hot carriers is the ability to transfer energy through nanogaps by tunneling, leading to electrically induced enhanced light emission with broadband photon generation, and when operated in reverse, photon detection through a significant open-circuit photo-voltage. These features originate from previously little appreciated concepts of electrically driven hot carrier processes.

## 2 Optically and electrically driven hot carrier processes: theory

Despite many experimental studies of plasmon-induced hot carriers, the understanding of the fundamental mechanisms underlying hot carrier generation and relaxation remains limited [36], [37], [38], [39], [40], [41]. It has been shown that the distribution of hot carriers generated through surface plasmon decay is distinct from those produced by direct excitation in several aspects. In both scenarios, the energy of the initial electron–hole pair matches the photon energy. However, in the case of surface plasmon decay, electrons tend to possess higher energies compared to the situation of direct excitation: electrons in Au under direct photoexcitation of sufficient energy are typically excited from the d-band with its edge ∼2.3 eV below the Fermi level to above the Fermi energy, while plasmon-induced carrier generation favors electron excitations from near the Fermi energy [33], [42]. It is also worth noting that hot carriers can be generated around the Fermi level in the same way as a plasmon decay would via intraband absorption in the absence of local field enhancement for photons below the band gap energy, albeit with lower efficiency. Moreover, plasmon-induced hot carriers are generated from the plasmonic near-field, which encompasses higher multipolar and dark modes [43]. For optical illumination only the bright plasmon mode are excited, whereas tunneling currents can excite optically dark modes. In this Section, we will discuss the hot carrier generation with different mechanisms [26], [44], [45], [46], [47] and the subsequent ultrafast relaxation dynamics.

### 2.1 Direct interband absorption

Direct transitions are highly sensitive to the band structure due to the selection rule of momentum conservation for both the initial and final electronic states [41]. For photons sufficiently energetic for interband transitions, the majority of the plasmon energy is utilized in exciting the electron from the *d*-shell to the Fermi level. Consequently, the generated electron is positioned not far above the Fermi level and generally lacks sufficient kinetic energy to tunnel through the barrier. Simultaneously, the hole in the *d*-shell exhibits a very low velocity, making it unable to reach the surface [45]. In copper and gold, the primary direct interband absorption occurs from the d-bands deep below the Fermi level to the conduction band, leading to high-energy holes and lower energy electrons. Conversely, in silver, absorption in the conduction band can generate high-energy electrons accompanied by lower energy holes. In aluminum, a continuous energy distribution of hot electrons and holes are generated, spanning from zero to plasmon energy.

Sundararaman et al. presented predictions concerning the immediate distributions of excited hot electrons and holes from plasmon decay prior to inelastic relaxation [41]. These predictions are derived from a quantized plasmon model that incorporates electronic structure details. Additionally, their focus is on interband absorptions, particularly those dominating at higher plasmon energies. These transitions are anticipated to be more responsive to the electronic structure compared to intraband transitions, which tend to dominate at lower plasmon energies and have been characterized within simplified models [27]. In Sundararaman et al.’s approach, they merge quantized plasmon modes derived from experimentally measured dielectric functions with electronic states obtained via first principles density functional theory. This enables them to compute the initial distribution of hot carriers in realistic materials. This method allows them to examine the role of the electronic structure of the metal on the resulting carrier distributions, independently of other factors such as geometry. Electronic band structure of the metal is introduced into the model by an approximation of the quasiparticle orbital $\psi_{qn}^{\sigma }\left(r\right)$ with eigenenergy *ɛ*_***q*** *n*_ inside the metal, which gives,

(2.1) $$\Psi_{\sigma }\left(r,t\right)=\underset{q,n}{\sum }\psi_{qn}^{\sigma }\left(r\right)e^{-\frac{i\varepsilon_{qn}t}{\hbar }}c_{qn}+\underset{q,n}{\sum }\psi_{qn}^{*,\sigma }\left(r\right)e^{i\varepsilon_{qn}t/\hbar }c_{qn}^{†}$$

where *c*_***q*** *n*_ and $c_{qn}^{†}$ denotes the Fermionic creation and annihilation operators for electrons with wave vector ***q*** and band index *n*. *σ* here represents the spinor orbital index. By applying Fermi’s golden rule, material-dependent hot carrier dynamics can be obtained. The calculation results reveal the sensitivity of the generated carrier profile to the details of the electronic band structure, particularly in relation to the position of *d* bands relative to the unoccupied states above the Fermi level in Ag, Cu, and Au. Cu and Au yield hot holes with considerably higher energies compared to the hot electrons. In the case of Ag, both hot holes and hot electrons with narrow energy distributions are produced. These results are also confirmed by Bernardi et al. using GW calculations [48].

### 2.2 Intraband absorption

In nanoscale systems, the electronic states become localized in space, and as a result, exact eigenstates for crystal momentum do not exist based on the uncertainty principle. This gives rise to a finite probability of direct plasmon decay into a hot electron-hole pair with a net crystal momentum. Such events can take place even for plasmons below the interband threshold energy, which is referred to as intraband absorption assisted by nano-scale geometrical effects. Compared to direct and phonon-assisted transitions which heavily rely on the electronic band structure of the metal, intraband absorption can be addressed by employing the free-electron-like model and including the geometry effects within the framework of quantum mechanics.

A theoretical approach can account for this effect by solving the Schrödinger equation of electronic states within the real geometry. Utilizing Fermi’s Golden Rule, optical matrix elements between these states are employed to calculate the transition rate induced by the plasmon.

Manjavacas et al. formulates a theoretical model to describe the plasmon-induced hot carrier excitation process and subsequently applies this model to both spherical Ag nanoparticles and nanoshells [27]. In this model, the conduction electrons of the metal are approximated as free particles within a finite spherical potential well. The process of plasmon-induced hot carrier generation is evaluated by applying Fermi’s golden rule. Hot carriers emerge through the interaction of both external and plasmon-induced electric potentials, which influence the conduction electrons of the metal. These interactions induce transitions from an initial electron state *ψ*_*i*_ below the Fermi level to a final state *ψ*_*f*_ above the Fermi level, with a hot electron–hole pair generated and a reduction of the plasmon occupation number by 1. The Hamiltonian of the above physical process is given by,

(2.2) $$H=\int \left[V\left(r,\omega \right)+V^{*}\left(r,\omega \right)\right]\underset{i,f}{\sum }\rho_{fi}\left(r\right)a_{f}^{†}a_{i}dr$$

Here, $V\left(r,\omega \right)$ denotes the total potential for the conduction electrons, $\rho_{fi}\left(r\right)=e\psi_{f}^{*}\left(r\right)\psi_{i}\left(r\right)$ and $a_{f,i}^{†}\left(a_{f,i}\right)$ is the creation and annihilation operator for the an electron(hole) in the system. The probability per unit time of generating an individual electron in the final state can be evaluated via Fermi’s golden rule,

(2.3) $$\begin{array}{ll} \Gamma_{e}\left(\varepsilon_{f},\omega \right) & =\frac{4}{\tau }\underset{i}{\sum }F\left(\varepsilon_{i}\right)\left[1-F\left(\varepsilon_{f}\right)\right]\\ & \;\times \left\{\frac{{\left|M_{fi}\left(\omega \right)\right|}^{2}}{{\left(\hbar \omega -\varepsilon_{f}+\varepsilon_{i}\right)}^{2}+\hbar^{2}\tau^{-2}}\right.\\ & \;\left.+,\frac{{\left|M_{if}^{*}\left(\omega \right)\right|}^{2}}{{\left(\hbar \omega +\varepsilon_{f}-\varepsilon_{i}\right)}^{2}+\hbar^{2}\tau^{-2}}\right\} \end{array}$$

where $F\left(\varepsilon_{i,f}\right)$ is the Fermi–Dirac distribution approximated at zero temperature, $M_{fi}=\int V\left(r,\omega \right)\rho_{fi}\left(r\right)dr$ represents the transition matrix element for the hot carrier generation process. The probability per unit time to generate a hole can be obtained in a similar way by interchanging the subscripts. The hot carriers will undergo various relaxation processes after being generated, including electron–electron and electron-surface scattering, and eventually release their energy to heat up the lattice through electron-phonon scattering. Here *τ* is the average lifetime that incorporates all these decay mechanisms of hot carriers. Essential to emphasize that this linewidth is fundamentally different from the plasmon damping *γ* in the Drude model ( $\varepsilon =\varepsilon_{b}-\omega_{p}^{2}/\omega \left(\omega +i\gamma \right)$ ), where *ɛ*_*b*_ is the background dielectric function and *ω*_*p*_ is the bulk plasma frequency.

The above model enables the calculation of the steady-state hot carrier distribution that emerges under continuous light illumination, offering a quantitative explanation specifically for scenarios involving pulsed illumination if the pulse duration is longer than hot carrier lifetime. In this model, hot electrons and holes are assumed to be localized and do not diffuse away from the location where they were excited. But it should be noted that the diffusive transport of hot carriers needs to be considered for extended nanostructures. In addition, simulating the time-dependent relaxation of excited carriers also becomes feasible. The hot carrier generation rate, which quantifies the efficiency exhibited by various plasmonic systems in generating advantageous hot carriers, can be estimated through,

(2.4) $$N_{e}\left(\varepsilon \right)=\hbar \omega_{sp}\underset{\varepsilon_{f}\geq \varepsilon }{\sum }\Gamma_{e}\left(\varepsilon_{f},\omega_{sp}\right)/P_{\text{absorb}}$$

(2.5) $$N_{h}\left(\varepsilon \right)=\hbar \omega_{sp}\underset{\varepsilon \leq \varepsilon_{f}}{\sum }\Gamma_{h}\left(\varepsilon_{f},\omega_{sp}\right)/P_{\text{absorb}}$$

where *ω*_*sp*_ denotes the plasmon resonance frequency where the nanoparticle reaches the maximal absorption, *P*_absorb_ is the total absorbed power of the nanoparticle. This model has been applied to many scenarios and provides quantitative agreement with experimental results [49].

Different theoretical approaches have been employed for calculating the electronic states, from the simple free-electron solution [50], [51], time dependent density functional theory that omits the atomic-scale structure in effecting the band structure [27], to detailed time dependent density functional theory accounting for the full band structure in small metal clusters on the size of 2–3 nm [52]. Through the substitution of free electron wave functions and energies with those derived from a density functional theory approach, Manjavacas et al. have also demonstrated that many-body effects cause only a marginal impact on carrier generation [27]. However, the hot carrier lifetime significantly influences both the production rate and the energies of the generated hot carriers (Larger sizes and shorter lifetimes lead to increased rates of hot carrier production, yet they correspond to lower energies of the generated hot carriers.). This theory has been applied to explain the energy distribution of nonlinear plasmon-assisted hot carrier generation [53] and the amplified hot electron generation in nanoparticle dimers with plasmonic hot spots [54].

While the free-electron models are highly effective in addressing geometry-assisted intraband absorptions, their applicability is confined to materials and plasmon energy ranges characterized by the presence of a free-electron conduction band. Hence, the majority of these studies are confined to examining silver, particularly below its interband threshold of 3.6 eV. For gold, copper and aluminum which exhibit numerous low energy direct absorptions, it is essential to take into account material dependent and phonon assisted process.

Due to the negligible momentum of photons and surface plasmons in comparison to the electrons’ crystal momentum, the direct decay of plasmons can only generate hot electron-hole pairs with a net zero crystal momentum. Such pairs of electronic states are typically accessible in the band structure of most metals above a certain interband threshold energy. In the scenario where the absorption and emission of phonons are involved in the plasmon decay, electronic states with different crystal momentum can be excited.

The phonon-assisted indirect optical absorption has been studied in indirect bandgap semiconductors by incorporating first principle *ab initio* calculations of phonon-assisted transitions and resistive losses in a semiconductor [55], [56], where the phonon-assisted absorption coefficient can be obtained using Fermi’s golden rule [68]:

(2.6) $$\begin{array}{ll} \alpha \left(\hbar \omega \right) & =\frac{8\pi^{2}e^{2}}{N_{k}N_{q}V_{\text{cell}}\omega cn_{r}\left(\omega \right)}\cdot \underset{vijkq}{\sum }{\left|\lambda \cdot \left(S_{1}+S_{2}\right)\right|}^{2}\\ & \;\cdot P\delta \left(\varepsilon_{j,k+q}-\varepsilon_{i,k}-\hbar \omega \pm \hbar \omega_{v,q}\right) \end{array}$$

where ***λ*** corresponds to photon polarization and $n_{r}\left(\omega \right)$ and *V*_cell_ is the refractive index of the material and effective cell volume, respectively. ***S***_1_ and ***S***_2_ are the generalized optical matrix elements corresponding to two different possible transition paths of indirect absorption process given by:

(2.7) $$S_{1}\left(k,q\right)=\underset{m}{\sum }\frac{v_{im}\left(k\right)g_{mj,v}\left(k,q\right)}{\varepsilon_{m,k}-\varepsilon_{i,k}-\hbar \omega +i\Gamma_{m,k}}$$

(2.8) $$S_{2}\left(k,q\right)=\underset{m}{\sum }\frac{v_{mj}\left(k+q\right)g_{im,v}\left(k,q\right)}{\varepsilon_{m,k+q}-\varepsilon_{i,k}\pm \hbar \omega_{v,q}+i\Gamma_{m,k+q}}$$

with *g* being the electron–phonon coupling matrix elements and ***v*** being the velocity.

By taking into account the contributions from “on-shell” intermediate states that correspond to sequential processes, Brown et al. extend the calculations of phonon-assisted transitions in the previous studies and generalized to metals [38]. Their findings reveal that above the optical band gap, direct transitions predominantly govern and result in the generation of hot carriers. Conversely, below the band gap, the production of hot carriers through phonon-assisted transitions is constrained by the presence of resistive losses, leading to reduced efficiency. They further proposed that Al holds significant promise as a versatile plasmonic hot carrier generator. It efficiently produces hot carriers across a wide frequency range and generates high-energy electrons and holes with equal probability. In comparison to noble metals, Al also boasts superior transport properties for high-energy holes.

The absorption assisted by electron–electron scattering is often omitted, partially due to the higher-order nature of the process, which involves two electrons and is therefore relatively weak and becomes evident only at high power levels. Such a process exhibits higher probability at higher photon frequencies as the scattering phase space scales with *ω* ^2^ for the involved two electrons and confirmed both from the experimental observation [57], [58] and theoretical calculations based on Fermi liquid theory [59] and permittivity response [60]. A comprehensive theoretical description of the electron–electron scattering process is intricate and should adhere to a thorough treatment within the framework of a Fermi liquid.

### 2.3 Landau damping

**Figure 2:**
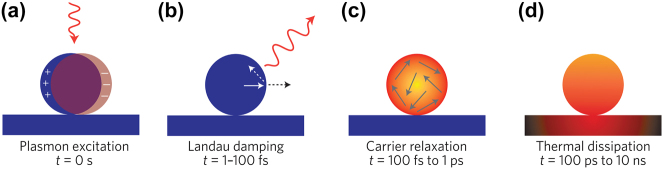
Hot carrier generation and relaxation dynamics. Hot carrier relaxation regimes at different times after initial photon excitation [26]. Reproduced with permission from the Springer Nature group.

An alternative way to maintain momentum conservation in the transition between two states with different wavevectors is through the surface collision effect, which occurs when the electron is reflected from the surface, and momentum is transferred between the electron and the entire metal body. The surface scattering rate was first proposed through a phenomenological theory with Γ_*s*_ ∼ *v*_*F*_/*a* where *v*_*F*_ is the Fermi velocity, *a* corresponds to the characteristics length of a particular metal particle. Such surface scattering-induced damping is shown to be related to the electrons’ Landau damping [61]–[63], where the field confinement in the nanostructure results in a large wavevector for localized surface plasmons resonances, facilitating the energy transfer between the electron and electromagnetic waves after the initial excitation of the hot carriers (Figure 2(a)). Khurgin et al. shown that confinement of electric fields in propagating surface plasmon polaritons enhances loss due to Landau damping, thereby limiting the extent of the confinement [63]. A comprehensive quantum-mechanical expression for the damping rate and the associated change in the imaginary part of the dielectric constant is proposed, confirming that surface collision damping not only increases the loss but also restricts the concentration of the electric field into localized regions. A similar formalism has also been employed by Shahbazyan [64], [65]. The entire Landau damping rate can be interpreted as the inverse of the time it takes for the average electron propagating along the field direction to reach the surface. For a surface plasmon polariton propagating along the interface plane between the metal and dielectric, the derived scattering rate agrees well with the intuitive interpretation provided by Kreibig and Vollmer [66].

In addition to the non-radiative damping for the creation of hot electron-hole pairs, a plasmon resonance can also be radiatively damped and generate far field photons, with the branching ratio determined by whether the nanostructure supports dark (subradiant) plasmon modes [67], [68]. The timescale for electron-hole pair creation during the plasmon damping ranges from 1 to 100 fs (Figure 2(b)), with the distribution of the hot electron–hole pairs relying on several factors, including the particle size, plasmon resonance energy and symmetry, and the carrier density of states within the material [27]. When the plasmon energy surpasses the interband transition, the majority of hot carriers will be hot holes at the upper edge of the metal d band, while electrons will be located near the Fermi level. Optimizing the hot carrier generation can be achieved through geometrically engineering the metallic nanoparticles to match the energy between the subradiant plasmon resonance and the large imaginary part of the permittivity.

The hot electrons produced from plasmon decay undergo rapid energy redistribution, dispersing their energy among numerous lower-energy electrons through processes like electron–electron scattering, including Auger transitions [69]. On the relaxation time scales around 100 fs to 1 ps, a Fermi–Dirac-like hot carrier distribution characterized by an effective electronic temperature can be formed, according to time-resolved studies [70], [71] (Figure 2(c)). Subsequent relaxation with the lattice occurs on a longer time scale via successive electron–phonon scattering events, spanning several picoseconds, and can be effectively described using the two-temperature model, accounting for the electronic and lattice temperatures [72]. Eventually, the heat is gradually dissipated to the surroundings over a time scale ranging from 100 ps to 10 ns, a duration determined by the specific material, nanoparticle geometry, and thermal conductivity (Figure 2(d)).

### 2.4 Various theoretical approaches

In addition to the various hot carrier excitation mechanisms mentioned above, hot electrons in metals have historically been investigated using multiple theoretical approaches [41], [73], which are briefly summarized in this section.

*Ab initio* electronic structure calculations have recently become viable for studying phonon-assisted transitions, and they have been employed to investigate the optical properties of indirect bandgap semiconductors [55], [56]. The *ab initio* tight binding approximation has been employed for calculating the electronic structure in thin films using Wannier functions [41]. Electron-temperature-dependent heat capacities and electron–phonon coupling coefficients of plasmonic metals can also be estimated through *ab initio* predictions [74]. Utilizing the *ab initio* metal dielectric function facilitates the calculation of the initial hot carrier distribution and the subsequent dynamics [37].

In metals, theoretical and computational studies of laser interactions have extensively utilized the two-temperature model proposed by Anisimov et al. [75], which uses two coupled nonlinear differential equations to describe the electronic and lattice temperature evolution both temporally and spatially when irradiated. After the initial relaxation of the generated hot carriers (∼100 fs), the hot carriers can be characterized by an elevated effective electronic temperature, where the dynamics can be well captured through the two-temperature model. Phenomenological estimates of electron and phonon coupling strengths, as well as relaxation times can be derived from the two-temperature model based on experiments [76]. By taking into account the non-thermal electrons during the thermalization process for the electrons and phonons, the extension of the two-temperature model is capable of exploring the contribution of non-thermal electrons in photocatalysis [77], [78].

In those structures governed by the ballistic and diffusive regimes that are either small or large when compared to the carrier mean free path, Boltzmann transport equation [79] or Monte Carlo methods [80] can be employed to investigate the transportation of plasmonic hot carriers. A thorough analysis of the transport of plasmonic hot carriers, considering all the effects such as the strong energy dependence of the electron lifetime and secondary electrons after multiple scattering events, requires treatment using the Boltzmann equation with spatial dependence. While such an analysis would be highly desirable for understanding the efficiency of plasmonic energy conversion, it poses significant computational challenges and thus is lacking.

### 2.5 Hot carrier relaxation dynamics

Previous studies have made substantial advancements in elucidating the significance of electron–electron and electron–phonon scattering during the relaxation of hot carriers [81], [82]. For example, sub-ps optical transient-reflection method and the first principles density functional theory have been employed to investigate the transient hot carrier lifetime for different metals. More complicated models have also been used to predict the lifetime of individual hot carriers within the independent electron picture, where exact matrix element calculations were applied [83], [84]. Calculations of these models are very challenging, limiting these approaches to either very large or very small nanoparticles with simple formalisms for the plasmon resonances [85]. In this regard, Liu et al. studied the relaxation dynamics of the hot carriers by extending the above model for hot carrier generation, and considering a pulsed excitation of the plasmon and investigating the hot carrier relaxation behavior towards thermal equilibrium [36]. To broaden the model’s scope and encompass nanoparticles of more realistic larger sizes, they introduce a parametrization for the electron–electron scattering process, where the rates are considered as a function of the energy difference between scattering states. Given the electronic energy bands of different metals, other structures can also be studied by employing this approach.

In Liu et al.’s approach, following plasmonic excitation, hot carriers relax via electron–electron, electron–photon, and electron–phonon scattering, with the time-dependent occupancies of electronic states determined through a coupled master equation. The electron–photon scattering refers to radiative decay of the plasmonic excitations through emission of a photon, in contrast to the non-radiative decay of plasmon-induced hot carriers through electron–electron and electron–phonon scattering. In the context of electrically driven plasmonic systems, the electron–photon relaxation mechanism is closely related to the below-threshold light emission process (described in detail in Sections 4.1 and 4.2), while the electron–electron and electron–phonon relaxation mechanisms dominate in the understanding of above-threshold emission (Sections 4.3 and 4.4). It is generally believed that energetic hot carriers can quickly thermalize via electron–electron scattering [86], [87], [88], before electron–phonon scattering becomes the dominant relaxation channel on the time scale of picoseconds [89], [90], as acoustic phonons have very low energies (∼meV) and it takes many subsequent electron-phonon collisions to change the energy of electrons. It is a reasonable approximation to assume a fixed electron–phonon relaxation timescale on the order of 1 ps for characterizing the electron thermalization process to the lattice and focus mainly on the electron–electron interaction within the few 100s of fs timescale of the hot carrier relaxation dynamics [91]. The electron–electron relaxation rate is then given according to Fermi’s Golden rule [36],

(2.9) $$\Gamma_{ij\to kl}^{ee}=\frac{4}{\tau }{\left|M_{ij\to kl}\right|}^{2}\frac{1}{{\left(\varepsilon_{i}+\varepsilon_{j}-\varepsilon_{k}-\varepsilon_{l}\right)}^{2}+\hbar^{2}\tau^{-2}}$$

where *ij* → *kl* denotes the initial and final state during an electron–electron scattering event. *M*_*ij*→*kl*_ is the transition matrix element corresponding to this process, which can be obtained by $M_{ij\to kl}=1/2\left|\left\langle i,j|V|k,l\right\rangle -\left\langle i,j|V|l,k\right\rangle \right|$ . Here *V* is the Yukawa potential induced by Thomas–Fermi screening of the Columb potential, $V=e^{-k_{TF}\left|r-r^{\prime }\right|}/\left|r-r^{\prime }\right|$ with *k*_*TF*_ ≈ 1 Bohr(Ag). *τ* refers to the natural linewidth of the hot carrier excitation and is chosen to be 1ps so that the plasmon decay induced initial hot carrier generation will not further increase after *τ*, which is different from hot carrier relaxation lifetime. This model thus can be implemented into more realistic systems by incorporating matrix elements for specific electronic band structures.

In addition to the pulsed excitation, for continuum optical excitation, it is also possible to build a model to describe the hot carrier distribution function under steady state and investigate the associated photoluminescence and electroluminescence processes. At high plasmon excitation rate (high photon intensity under optical excitation or electrical current excitation), hot carriers generated by plasmon decay can lack adequate time to return to their initial conditions before the subsequent plasmon excitation which further generates additional hot carriers. This process leads to a steady state nonequilibrium hot carrier distribution within the material. Such a distribution can be described using the average hot carrier distribution energy,

(2.10) $$k_{B}T_{hc}={\bar{E}}_{hc}=\left\langle \frac{\int \varepsilon d\varepsilon p_{E}\left(t,\varepsilon \right)}{\int d\varepsilon p_{E}\left(t,\varepsilon \right)}\right\rangle$$

where *p*_*E*_(*t*, *ɛ*) is the population distribution of carriers at time *t* after hot carrier generation and energy *ɛ* taken as the energy difference relative to the Fermi level, *ɛ* = *E* − *E*_*F*_. *T*_*hc*_ can be understood as the effective hot carrier temperature that characterizes the average hot carrier energy. Note here that the defined effective temperature is distinct from the Fermi temperature corresponding to the energy of the Fermi level divided by the Boltzmann constant, where the average energy at 0 K for the entire Fermi Sea is 3*E*_*f*_/5. For simplicity, consider a three-level system consisting of $\left|g\right\rangle$ , $\left|e_{1}\right\rangle$ and $\left|e_{2}\right\rangle$ representing the ground state, the first, and second excited state with $\left|e_{2}\right\rangle$ having higher energy, respectively. Electrons will be first excited to $\left|e_{2}\right\rangle$ then decays to $\left|e_{1}\right\rangle$ with a characteristic decay time *τ*_2_, which subsequently decays to the ground state from $\left|e_{1}\right\rangle$ with a characteristic decay time *τ*_1_. In steady state, the populations of $\left|e_{1}\right\rangle$ and $\left|e_{2}\right\rangle$ can be calculated as [36],

(2.11) $$p_{2}=\frac{e^{-\frac{\beta }{I\tau_{2}}}}{1-e^{-\frac{\beta }{I\tau_{2}}}}$$

(2.12) $$p_{1}=r_{21}\frac{e^{-\frac{\beta }{I\tau_{1}}}}{1-e^{-\frac{\beta }{I\tau_{1}}}}$$

where *β* = *ℏω*/*σ* represents the absorption ability under electrical or optical excitation with *σ* being the absorption cross section. *r*_21_ is the relative decay rate from $\left|e_{2}\right\rangle$ to $\left|e_{1}\right\rangle$ . *I* here is the intensity of the external excitation, either electrically or optically, manifested through optically injected photons or electrically injected charges. This phenomenological model can be used to explain the plasmon heating induced hot carrier generation [92] and the giant photon up-conversion when combining electrical and optical excitation [19].

To summarize, the attempts to develop semi-classical and quantum theoretical approaches to describe hot carrier processes in plasmonic nanostructures have yield great insights which are crucial to the understanding of experimental results in this field. However, quantitative models incorporating the full details of hot carrier generation and relaxation dynamics of realistic plasmonic materials under different excitations still need to be established. These models could serve as important roles to address discrepancies in plasmonic experiments and provide a predictive platform for the design and optimization of plasmonic devices in various applications.

## 3 Plasmonic hot carrier detection and imaging: experimental techniques

Experimental studies of hot carriers are challenging on both temporal and spatial scales: the typical lifetime of hot electrons and hot holes spans from the few-fs to lower ps range, and the mean free path of hot carriers only extends to a few 10s of nm. To visualize and track the transport and dynamics of hot carriers, experimental tools are needed with simultaneously high spatial and time resolutions. In this Section, we discuss several important experimental advances that have been made in recent years to collect, detect, and image hot carriers.

### 3.1 Nanometer-scale energy barrier for hot carrier collection

**Figure 3:**
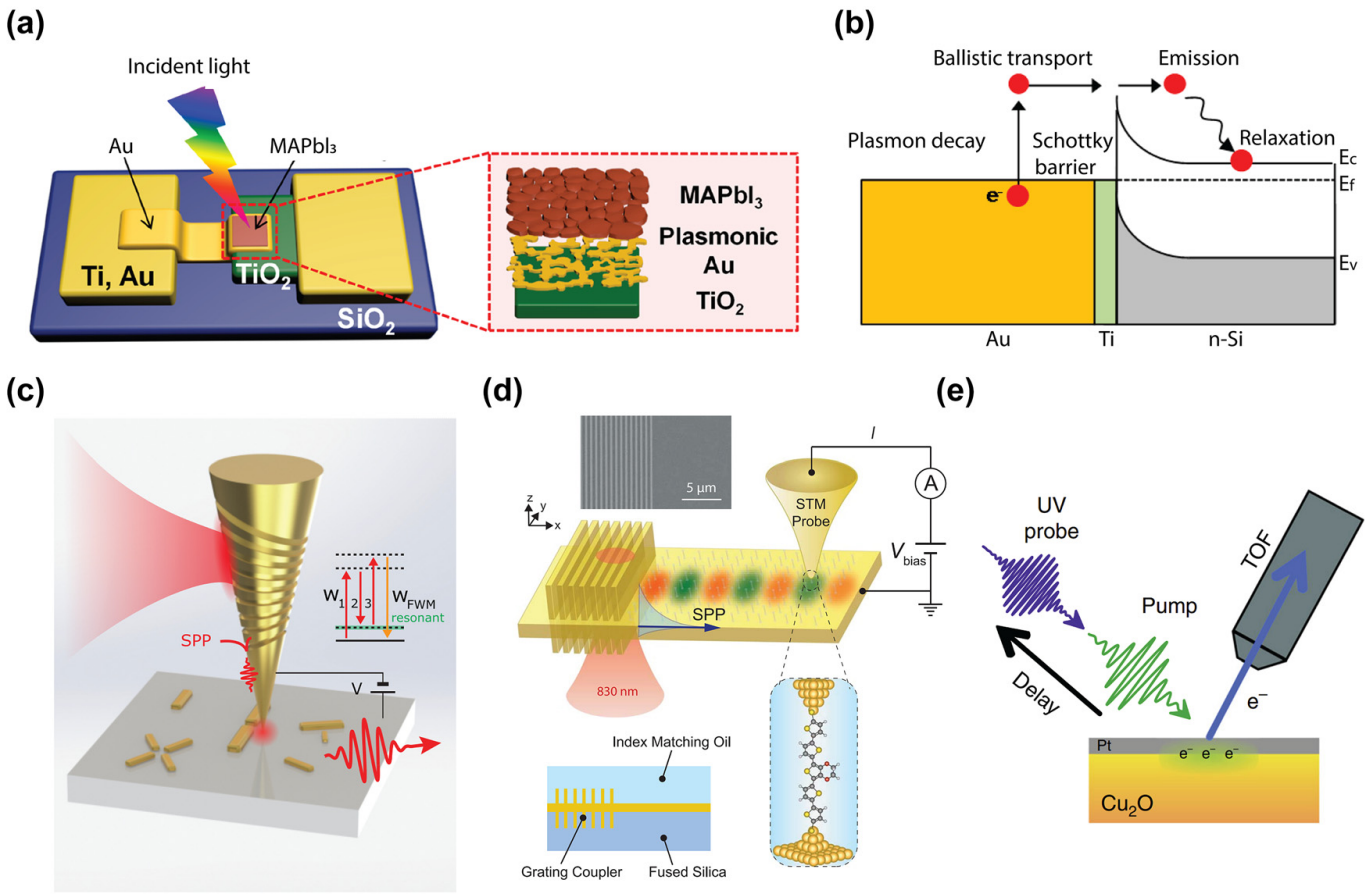
Experimental techniques for hot carrier detection and imaging. (a) Illustration of the MAPbI3/Au/TiO_2_ nanodiode configuration. The inner structure can be seen in the enlarged view of the active area [93]. Reproduced with permission from the American Chemical Society. (b) A plasmon-induced hot electron can undergo ballistic transport to the Schottky barrier interface, leading to the tunneling into the semiconductor [96]. Reproduced with permission from the American Chemical Society. (c) A pair of femtosecond pulses of variable time delay are grating coupled and focused at an STM apex, which can be used as a ultrafast nanoimaging tool [112]. Reproduced with permission from Springer Nature Group. (d) The hot carrier distribution induced by the propagating SPPs from the grating is mapped by the single molecule junction based on the STM probe [119]. Reproduced with permission from American Association for the Advancement of Science. (e) A schematic illustration depicts the application of time-resolved two-photon photoemission spectroscopy to Cu_2_O [122]. Reproduced with permission from Springer Nature Group.

Upon generation, hot carriers undergo rapid thermalization, rendering their collection challenging and impeding their practical utilization. Therefore, a Schottky diode, comprised of a metal–semiconductor junction with a Schottky barrier height, proves advantageous for capturing the excited hot carriers before they dissipate their energy. The Schottky barrier height and internal band bending within the semiconductor facilitate the separation of hot carriers, thereby getting rid of low-energy carriers and obstructing the reverse diffusion of the transferred hot carriers. Park et al. fabricated a perovskite plasmonic nanodiode consisting of MAPbI_3_ layers covering a plasmonic-Au/TiO2 Schottky junction (Figure 3(a)), with randomly connected Au nano islands deposited on a TiO_2_ layer serving as the source for hot carrier flux generation [93]. The measured incident photon-to-electron conversion efficiency and the short-circuit photocurrent exhibit a remarkable enhancement in the light-to-electrical conversion. Femtosecond transient absorption spectroscopy also reveals extended lifetimes of hot electrons in plasmonic Au structures combined with MAPbI_3_. A similar structure based on p-type GaN combined with gold nanoparticles had been investigated by DuChene et al., showing photoelectrochemical properties that align with the injection of hot holes from Au nanoparticles into p-GaN following plasmon excitation [94]. The straightforward fabrication and integration process of Schottky diodes permits the adoption of various materials and geometries, encompassing nonmetallic plasmonic materials [95] and three-dimensional Schottky structures [96] (Figure 3(b)). Furthermore, the electrical access for the Schottky diodes enables engineering to harvest hot-electrons derived from plasmonic excitations in electrically active plasmonic devices, showing potential for photovoltaic applications based on hot carriers [97].

### 3.2 Electron microscopies for hot electron imaging

To image the hot carriers generated by surface plasmon decay, experimental techniques with abilities to characterize the extreme near field of surface plasmons with high spatial resolution are needed. Leveraging the high resolution of the electron microscopy system, Nelayah et al. performed electron energy-loss spectroscopy (EELS) experiments in the context of a scanning transmission electron microscope [98]. A distinctive feature of this instrument is its ability to simultaneously detect multiple signals for both energy-loss spectra and the corresponding projected mass signal at every probe position during the scanning process. By utilizing electron beams instead of photons to record plasmon maps within the near infrared, visible, and ultraviolet spectrum, high spatial resolution imaging of plasmonic modes is possible. Nicoletti et al. used a similar electron energy-loss spectrum imaging to obtain the three-dimensional images of the localized surface plasmons for individual Ag nanocubes, providing experimental evidence of higher-energy mode hybridization [99]. Frank et al. visualized the propagation of short-range surface plasmon polaritons and sub femtosecond dynamics using normal-incidence two-photon photoemission electron microscopy for atomically flat single-crystalline gold platelets on silicon substrates [100]. Further imaging spectroscopy that involves electron excitation also includes scanning electron microscope [101], [102], transmission electron microscope [103], and cathodoluminescence spectroscopy [104], [105], [106] which measures the far field emitted photons, showing a spatial resolution down to sub-10 nm.

### 3.3 Scanning probe microscopies from electronically coherent to hot carrier dynamics imaging

Other powerful approaches for nanoscale or even atomic-scale plasmon and hot carrier dynamics imaging are based on scanning near-field microscopies. Here, a nanoscale metal tip provides both topograpic information and nano-scale optical field localization when operated in atomic-force or scanning tunnelling microscope mode.

Examples include near-field enhancement and work function differences that arise from charge distribution of surface plasmon polaritons (SPPs) which are accessible through the scanning near-field optical microscope [107] and Kelvin probe force microscopy [108], [109], [110], [111], with spatial resolution as high as 2 nm.

Of particular interest is the ability to image the electronic coherent dynamics of localized surface plasmons following femtosecond pulsed excitation. Following the initial elementary steps of the light–matter interaction, these coherent electronic processes precede the hot electron generation. This has been enabled in the implementation of time-resolved nonlinear second-harmonic generation (SHG) and four-wave mixing (FWM) with scanning probe implementation for simultaneous few-fs temporal and nanometer spatial resolution. In order to provide both nanoscale resolution and contrast, Kravtsov et al. used adiabatic nanofocusing in combination with pulse shaping and control to achieve a femtosecond light source at the apex of the scanning probe tip [112]. This allowed for spatially resolved imaging of the few-fs coherent dynamics of plasmonic nanostructures, resolving heterogeneity and coupling, with the dynamics controlled by intrinsic Drude damping and extrinsic radiative decay (Figure 3(c)).

Imaging and probing the subsequent hot carrier generation during the decay of surface plasmons can be achieved both in the spectral and temporal domain. Examples include the quantification of the electron temperature and relaxation rate from the analysis and power dependence of incoherent hot electron photoluminescence and FWM efficiency, respectively [113]. As discussed above, the carrier relaxation is a combination of different, in part competing, elementary processes of electron–electron and electron-phonon scattering with both affected by the type of metal as well as finite-size effects as dimensions shrink to the atomic scale. Through active modification of the different relaxation pathways, additional information can be gained aiding in understanding their relative contributions. Examples include the study of the decay of localized surface plasmons combining AFM-based near-field imaging with scanning tunneling microscopy (STM) based techniques coupling a tunneling pathway to the SPP excitation. In the example of a LSPR excited in a Au nanotip in close proximity to a mirror substrate, the transition of classical electromagnetic to quantum coupling could be demonstrated with associated changes in spectral position, LSPR lifetime, and amplitude [114]. This work demonstrated the exceptionally short few-fs radiative lifetime of LSPRs, highlighting their distinct light emission behavior compared to atomic or molecular emission.

STM itself can be extended into the time domain when combined with THz spectroscopy. Lock et al. presented findings from variable temperature and voltage measurements that explore the nonlocal manipulation of adsorbed molecules on the Si surface using a STM, showing that a two-dimensional diffusive model can be used to explain the hot electron dynamics on the 10 nm scale [115]. Sabanés et al. shown that ultrafast electronic heating and transient thermionic tunneling can be investigated in a metallic photoexcited tunnel junction. Phase-resolved sampling of broadband THz pulses allows the real time monitoring of the competition between ultrafast thermal and nonthermal photocurrents, therefore revealing the dominant role of nonthermal electron distribution and delayed tunneling of thermalized hot electron in the in the hot electron tunneling from a laser excited STM tip [116]. Another technique which is referred to as a scanning noise microscope can also be used to investigate the ultrafast dynamics of the nonlocal hot carriers. Weng et al. realized electronic nano thermometry by measuring the current shot noise linked to the ultrafast hot electron dynamics. By using a non-contact W tip, they mapped the hot carrier distribution in real space before carriers thermalized to the lattice by probing the electrical shot noise in the extreme near field [117]. Similarly, a Schottky diode-terminated tapered tip of nanoscale dimensions is employed for adiabatic focusing of surface plasmons with a plasmon-to-hot-carrier conversion efficiency exceeding 30 %, showing the potential for a novel nanoscopy technique [118]. More recently, Reddy et al. performed hot carrier transport measurements through single-molecule junctions (Figure 3(d)), realized through a single molecule sandwiched between an ultrathin plasmonic Au film and a scanning probe tip [119]. The gold thin film serves as a source supporting propagation surface plasmons, and the single molecule junction works as an energy filter to determine the quantification of plasmonic hot carrier energy distribution at steady state. This work shows that the Landau damping is the dominant hot carrier generation mechanism in plasmonic nanostructures with strong field confinement effects.

### 3.4 Photoemission spectroscopy

The fundamental timescale associated with the excitation of electronic states in metals is very short, typically on the order of a few femtoseconds (fs). Therefore, the time-resolved two-photon photoemission [120], [121] provides a possible way in probing the short lifetime (Figure 3(e)) [122]. In a time-resolved two-photon photoemission experiment, an optically induced electronic excitation’s lifetime is assessed in the time domain by detecting the signal decrease based on the time delay between a pump and a probe laser pulse. The ultrafast dynamics of excited carriers is probed at low excitation density and pump-pulse intensities lies in the few μJ/cm^2^ range, where the scattering between excited electron and the lattice heat up effect is negligible, so that the photoemission is limited to the sequential absorption of two photons. Following the initial time-resolved studies on image potential states on Ag(100) [123], further advancements have been achieved in understanding electron localization and solvation at interfaces [124], [125], [126] and adsorbate-surface hybrid systems [127], [128]. There are also parallel works for investigating the carrier dynamics in semiconductors using time-resolved two-photon photoemission such as GaAs [129] and TiO_2_(110) [130]. The Heisenberg equations of motion are utilized to model the experimental data, offering qualitative insights into the thermalization and cooling processes of hot electrons [131]. In contrast to studies of excited electronic states in metals, the majority of time-resolved two-photon photoemission experiments conducted on semiconductors concentrate on ensemble dynamics following intense optical excitation [132], [133].

Conversely, in time-resolved photoelectron spectroscopy [134], [135], a higher intensity pump-pulse is employed (mJ/cm^2^), resulting in an increased density of the hot carriers that lead to demagnetization [136], desorption of absorbed molecules [137] and a notable rise in lattice temperature. After the mid-2000s, time-resolved photoelectron spectroscopy has been routinely used for probing non equilibrium dynamics of solids [138], [139], [140], [141]. These studies show a significant level of maturity in this field. However, the interpretation of experimental results has been a long-standing debate in the past, where a specific challenge involves the intricate task of distinguishing between the various mechanisms implicated in the generation and decay of hot electrons.

## 4 Electrically driven light emission in molecular scale plasmonic nanostructures

**Figure 4:**
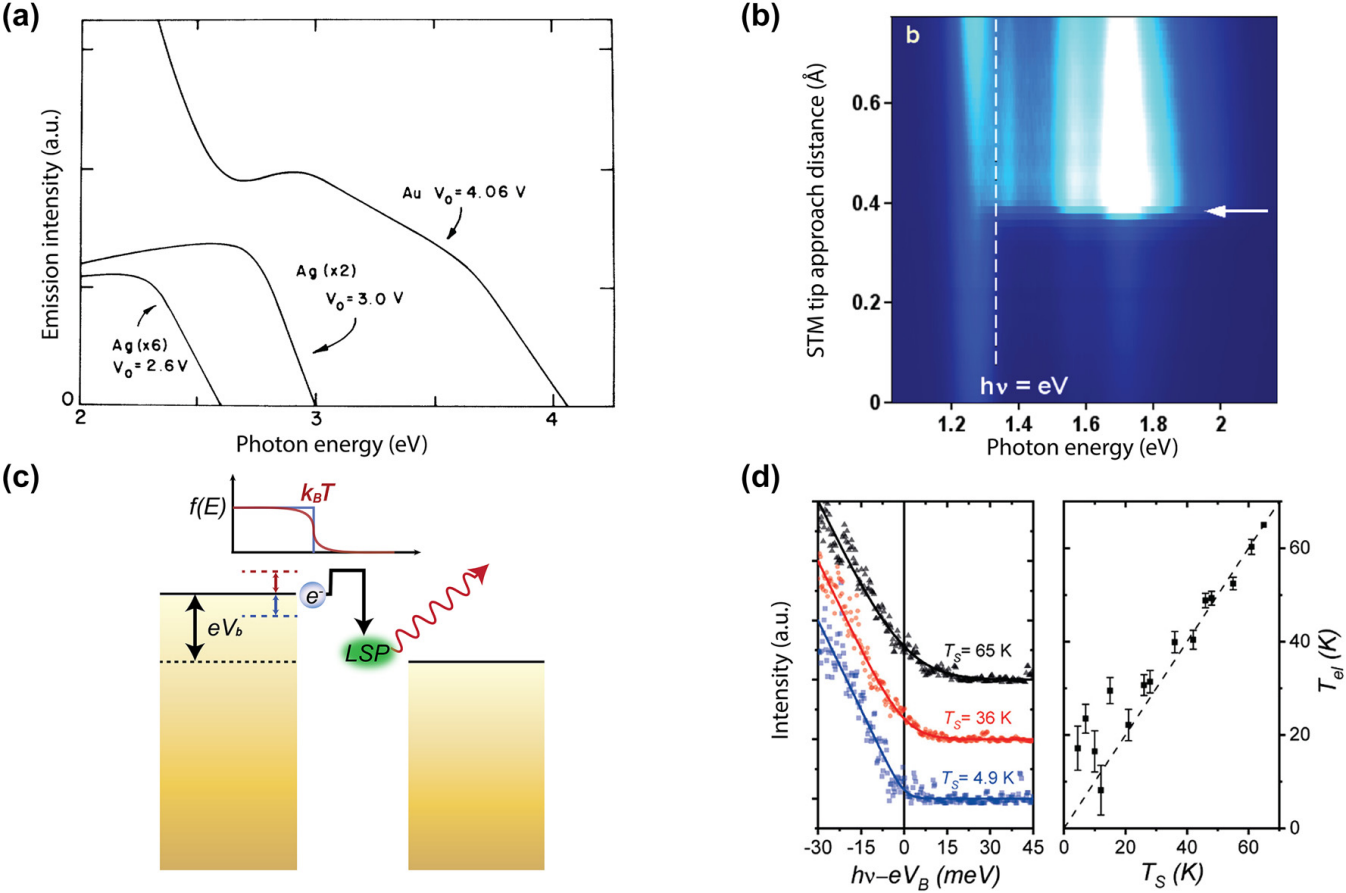
Below-threshold light emission in plasmonic nanostructures due to inelastic electron tunneling and thermally broadening induced overbias emission (a) light emission spectra from electrically biased Au and Ag tunnel junction at 77 K, an energy cutoff equal to the applied bias can be clearly seen [18]. (b) Light emission spectra map from a biased STM junction when varying the tip surface distance [147]. (c) An electron can gain excess energy from the thermal broadening of the Fermi–Dirac distribution, creating a higher energy LSP which leads to the overbias emission [148]. (a)–(c) are reproduced with permission from the American Physical Society. (d) Electronic temperatures obtained from fitting the tail at the energy cutoff position of the spectral data, plotted together with the junction temperature in the right panel [160]. Reproduced with permission from the Springer Nature Group.

Light emission from electrically biased tunnel junctions has been a well-known phenomenon since the notable work in the 1970s by Lambe and McCarthy [18] (Figure 4(a)). The underlying mechanism is that LSPRs adjacent to the atomic sized tunneling nanostructure can be excited inelastically by the tunneling electrons and subsequently experiences rapid radiative decay into far field photon emission. One key feature of inelastic tunneling induced light emission is its broadband spectrum and at photon energies *ℏω* less than the nominal single electron energy corresponding to the applied voltage (i.e., *ℏω* ≤ eV, also called below-threshold light emission). Interestingly, several pioneering works [142], [143], [144], [145], [146], [147] performed by scanning tunneling microscopy and nanofabricated tunnel junctions have reported the observation of above-threshold light emission, i.e., emission of photons having energies extending to double or triple of the single electron level in contradiction with the simple single electron picture in previous studies (Figure 4(b)). These observations have invoked significant interest in understanding the fundamental energy conversion physical mechanisms under different experimental conditions. From the perspective of practical applications, such emission can potentially provide novel miniaturized photoemitters controlled by purely electrical means and could be engineered down to the atomic scale. In this section, we will review previous works which will be sorted by their respective physical mechanisms, with the focus mainly on a recently proposed electrically driven hot carrier-based process [148]. Potential applications regarding the molecular scale electroluminescence and quantum light sources based on electrically driven plasmonic tunnel junctions will also be discussed later in this section.

### 4.1 Basics of inelastic electron tunneling and below-threshold light emission: low current limit and finite temperature effects

A tunnel junction is an intrinsically atomic and molecular scale nanostructure, and when it is biased electrically, charges flow via quantum tunneling through the potential barrier. In this process, most electrons elastically transport through the junction without losing their energy. A small fraction (<10 %) of the tunneling electrons, undergo inelastically tunneling and lose energy to excite other excitations in the junction such as localized surface plasmons, molecular vibrations, or phonons. In particular, LSPR’s can subsequently decay radiatively to generate photons. In the presence of molecules in the tunnel junction, inelastic electrons can interact with the vibrational modes in the molecules, pumping the local vibrations to higher energy states, leading to potential molecular electroluminescence. In theory, such inelastic process and subsequent photon conversion can be described as a Purcell effect controlled by the near-field local electromagnetic density of states in the tunneling junction. In experiment, transduction of mid-infrared (MIR) light from the molecular vibrations is possible to produce upconverted signal as enhanced visible luminescence, thus allowing the single-molecule MIR detection [149].

The emission spectrum from these electrically driven tunnel junctions and ultra-thin plasmonic gaps is determined in combination with the detailed atomic scale morphology and the detailed condition of inelastic electron tunneling. The former acts as a plasmonic nanoantenna that imprints the surface plasmon mode into the spectrum, which yields,

(4.1) $$U\left(\hbar \omega ,V_{b}\right)=\rho \left(\hbar \omega \right)\cdot S_{\text{iet}}\left(\hbar \omega ,V_{b}\right)$$

where $U\left(\hbar \omega ,V_{b}\right)$ is the light emission spectrum that is dependent on the photon energy *ℏω* and the electrical bias *V*_*b*_. *S*_iet_ is the function related to the inelastic electron tunneling process which we will discuss later.

We first examine the zero temperature (*T* = 0 K) and low current limit of the light emission from inelastic tunneling electrons. The tunneling behavior of electrons leads to a quantum shot noise, with the frequency cut off set by the applied bias and temperature [150]. The current fluctuation can transfer energy out from a biased quantum point contact [151] and it has been shown by Gustavsson et al. that the process can be mediated by photons at high-frequency current fluctuations [152]. Therefore, by driving the current noise into the optical frequency regime, far field emission in the visible can be observed [153]. At zero temperature, *S*_iet_ in Eq. (4.1) can be expressed by current noise spectral intensity [151], [154], [155], [156],

(4.2) $$\begin{array}{ll} S_{\text{iet}}\left(\hbar \omega ,V_{b}\right) & =G_{0}\underset{i}{\sum }T_{i}\left(1-T_{i}\right)\left[W,\left(\hbar \omega -eV_{b}\right)\right.\\ & \;\left.+,W,\left(\hbar \omega +eV_{b}\right)\right]+2G_{0}\underset{i}{\sum }T_{i}^{2}W\left(\hbar \omega \right) \end{array}$$

where *G*_0_ is the conductance quantum *G*_0_ = 2*e* ^2^/*h* and *k*_*B*_ is the Boltzmann constant. *T*_*i*_ is the transmission probability of the *i*th conducting channel with the total junction conductance given by *G* = *G*_0_∑_*i*_ *T*_*i*_. $W\left(\varepsilon \right)$ is a function expressed by $W\left(\varepsilon \right)=\varepsilon {\left(e^{\varepsilon /k_{B}T}-1\right)}^{-1}$ . If the conductance and the tunneling current are both very small, the higher order *T*_*i*_ term is negligible and the $S_{\text{iet}}\left(\hbar \omega ,V_{b}\right)$ can be simplified as,

(4.3) $$S_{\text{iet}}\left(\hbar \omega ,V_{b}\right)=G_{0}\underset{i}{\sum }T_{i}\left(1-T_{i}\right)W\left(\hbar \omega -eV_{b}\right)$$

with the noise spectra cutoff frequency *ω*_cutoff_ set by *ω*_cutoff_ = *eV*_*b*_/*ℏ*. Therefore, in inelastic tunneling induced light emission, the upper limit of the emission is bounded by the driven voltage *eV*_*b*_.

This below-threshold light emission is often observed in STM based tunnel junctions. Sparks and Rutledge studied a series of vertical Au-oxide-Al biased light emitting tunnel junctions with ranging oxide thickness, showing the cutoff emission scattering off the junction slow mode as the dominant mode [157]. Schull et al. showed that in an ultrahigh vacuum at 6.8 K, an STM tunnel junction formed by Au(111) surface and chemically etched W tips can emit below-threshold light (Figure 1(c)) when the tip sample distance is well above single atom contact [147]. In van der Waals quantum tunneling devices realized through 2D material heterostructures such as gold, hexagonal boron nitride (hBN) and graphene, inelastic electron tunneling can also result in photon emission with photon energy below the applied bias. A resonant enhanced photon emission rate can be further achieved by coupling these heterostructures with designed optical nanocube antennas [158].

At elevated temperatures, more pronounced current fluctuation can break the strict energy limit in the above-mentioned below-threshold light emission (Figure 4(c)). Kalathingal et al. performed a scaling analysis to the measured emission spectrum from a STM junction by incorporating a theoretical model that takes into account the finite temperature contribution during the quantum transport process [159] (Figure 4(d)). Photon emission at energies higher than the quantum cutoff (*eV*_*b*_) can be well explained by the junction conductance variation with *V*_*b*_. Similar phenomena had also been observed for photon emissions from Au/Au and PtIr/Au STM junctions under ambient conditions, where the high energy cutoff breaking the quantum relationship *ℏω*_cutoff_ = *eV*_*b*_ was attributed to local increase of the temperature of the sample surface (surface-enhanced phenomena) [144]. Such a tunnelling electroluminescence emission edge can be utilized as a thermometer to accurately measure the electronic temperature through the spectral decay shape beyond *eV*_*b*_, yielding a 30 K temperature difference between electron and lattice in a STM Ag/Au junction with tunneling current larger than 15 nA [160]. Furthermore, a finite lifetime of the excited states derived from time-energy uncertainty was used to correct the spectra when the thermal broadening of the Fermi distribution cannot be used alone to account for the spectra exceeding the electrical energy [161].

### 4.2 Multielectron coherent tunneling process and above-threshold light emission

When increasing the current, higher order tunneling terms in Eq. (4.2) must be considered, and the emission spectrum is no longer strictly limited by the voltage bias defined energy threshold. The probability that two or more electrons coherently tunnel through the junction is no longer negligible compared to the single inelastic tunneling events, leading to the emission of the so called “2e”, “3e” photons etc. To date there are several theoretical models developed to account for the multielectron coherent tunneling, with variations in the detailed picture of tunneling process.

**Figure 5:**
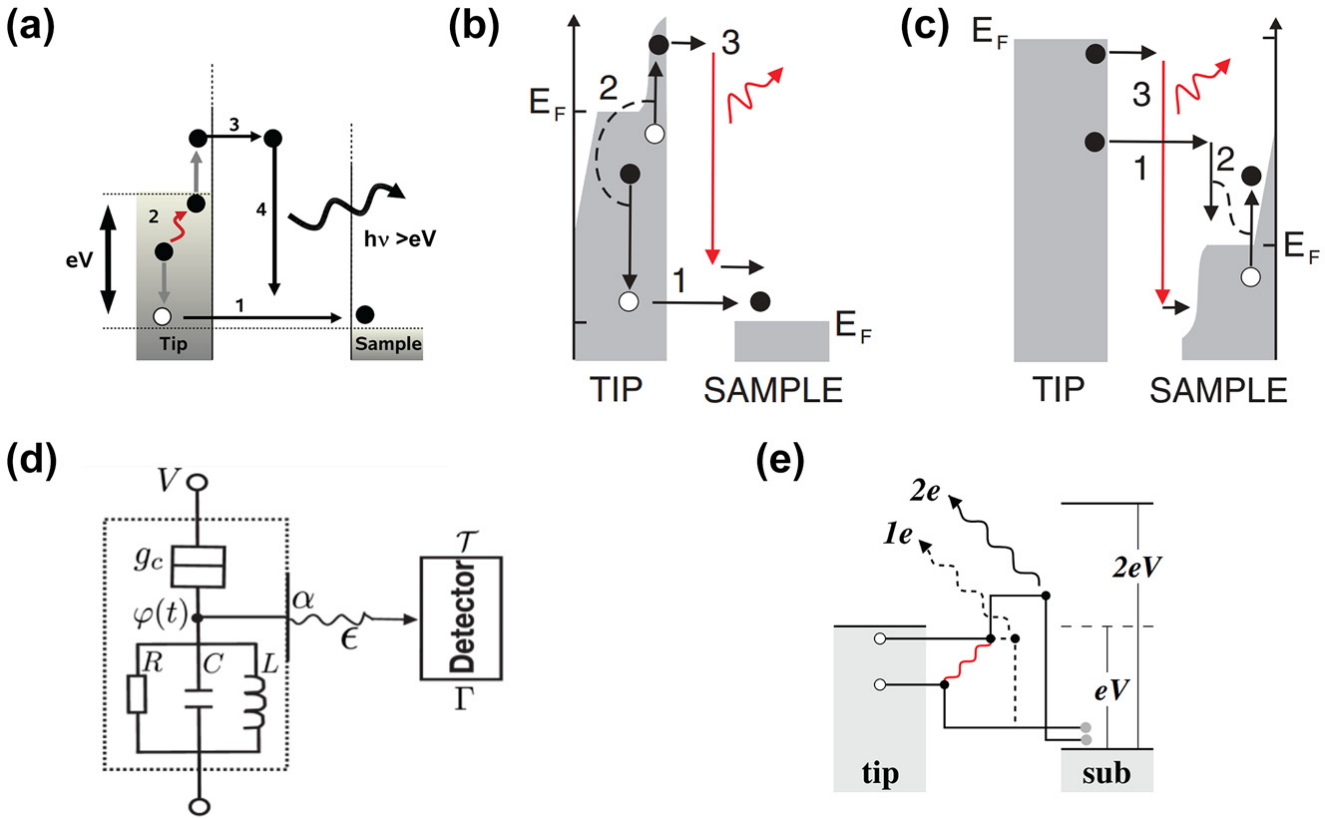
Multielectron coherent tunneling induced above-threshold light emission (a) illustration of the hot-hole mechanism producing the 2e light emission [147]. (b) A hot hole created by elastic tunneling below the Fermi level can go through a cascade and produce a high energy electron above Fermi level, which leads to the overbias photon through inelastic tunneling [162]. (c) A hot electron can tunnel into the sample, where a subsequent cascade process generates hot holes. The electron–hole recombination gives rise to the 2e light emission [162]. (d) The effective electromagnetic circuit model describing the light emission process: SPP damping and photon emission is mimicked through a LRC circuit [163]. (e) One and two electron coherent tunneling for the 1e and 2e light emission [165]. All figures are reproduced with permission from the American Physical Society.

Hoffmann and Berndt proposed an Auger-like process where two electrons exchange their energy via Coulomb interaction during their simultaneous tunneling, which is called hot-electron–hole cascade mechanism [143]. The hole cascading deep below the Fermi sea provides the excess energy to the hot electrons, which then subsequently excites the LSPR, leading to 2e emission (Figure 5(a)) [147]. The same group performed a more detailed study showing the transition from 1e to 2e light emission by altering the STM junction conductance close to the single atom contact. They found that the power law dependence of 1e, 2e light, and the tunneling current on logarithmic scale was drastically different (Figure 5(b)) [162]. Later, they determined the hot electron distribution at the tip area based on the shape of the emission spectra, yielding a deviation from the Fermi–Dirac function with a high-energy tail above 2000 K. An alternative process was proposed in which an injection of a hot electron results in a cascade which produces a hot hole to recombine with an inelastically tunneled electron and generate 2e light (Figure 5(c)) [162].

Xu et al. proposed a model for the above-threshold light emission in analogy to the treatment of the electromagnetic environment in a mesoscopic circuit (Figure 5(d)). The 2e process and the subsequent scattering with the localized plasmon mode is modelled by an effective LC circuit, with the above-threshold light emission originated from non-Gaussian part of the current noise [163]. Later, they generalized this model to finite tempera-ture, where thermal smearing cannot be ignored in emission spectra [164]. At almost the same time, Kaasbjerg and Nitzan developed a theory for the above-threshold light emission by calculating higher order of the quantum noise and link it with plasmon induced electron–electron interaction [165], as can be seen in Figure 5(e).

**Figure 6:**
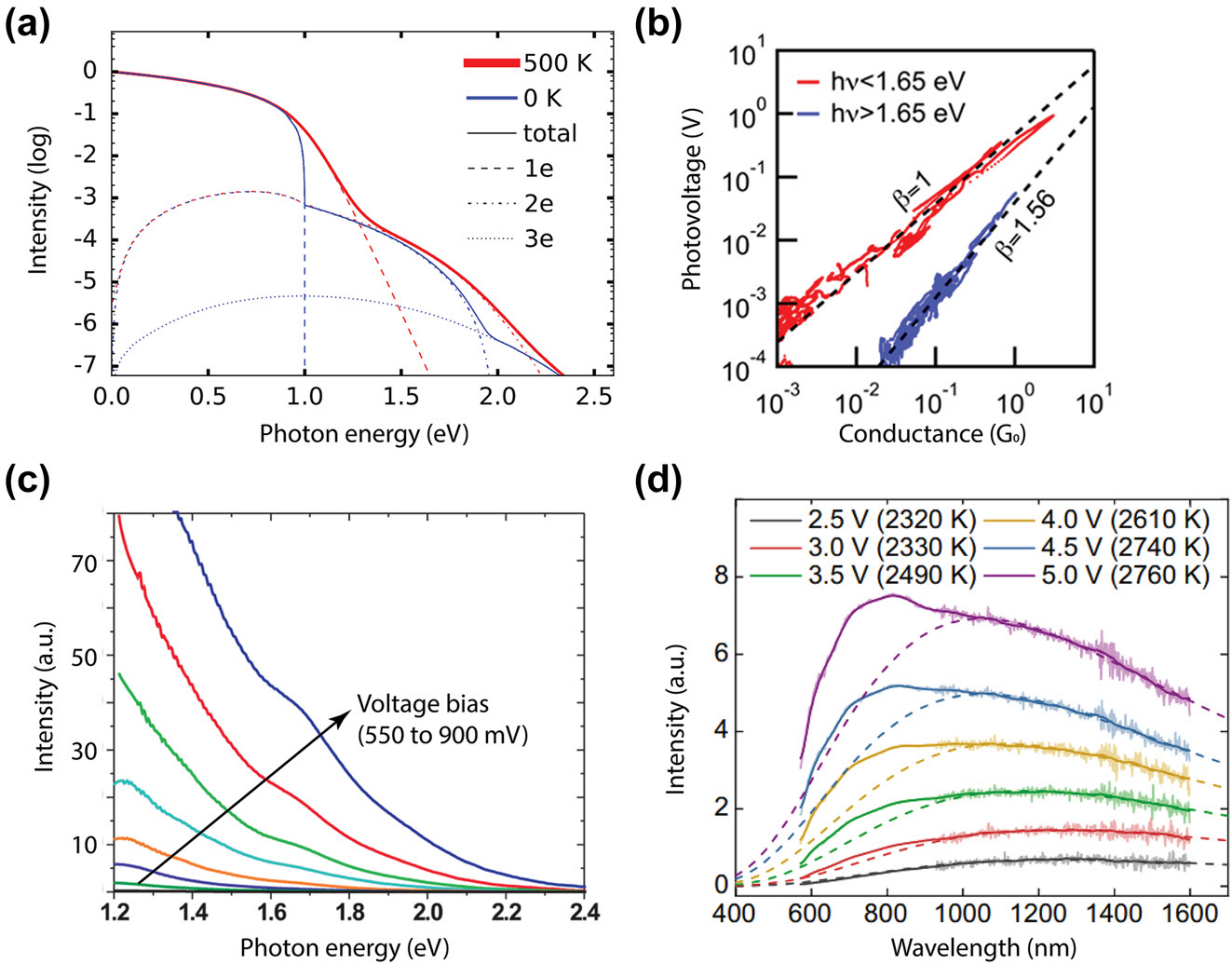
Experimental results for multielectron tunneling and blackbody-like light emission. (a) Calculated light emission spectra of multielectron cotunneling processes at *T* = 0 and 500 K. Contributions from 1e, 2e, and 3e processes are indicated by dashed, dash-dotted, and dotted lines, respectively. The sharp turns occurring at 1e, 2e, and 3e position are smeared out at elevated temperatures [146]. Reproduced with permission from the American Physical Society. (b) Traces of measured photovoltage (proportional to the emission intensity) versus the STM junction conductance for both below- and above-threshold emission [166]. Reproduced with permission from the American Chemical Society. (c) Emission spectra at different biases from a planar electromigrated junction, with giant overbias emission [145]. Reproduced with permission from the American Chemical Society. (d) Emission spectra from a planar graphene tunnel contact, which resemble the blackbody radiation spectra (dashed line) but with an added spectral weight [170]. Reproduced with permission from the American Physical Society.

More recently, Peters et al. performed a measurement of the light emission from an STM based junction structure which reveal a kink-like feature at *ℏω* = *eV*_*b*_ and 2*eV*_*b*_, indicating the emission of 1e, 2e, and 3e light from the quantized multielectron tunneling process [146] (Figure 6(a)). By combining the above mentioned two models, they proposed a model for multielectron coherent scattering with the local plasmon resonance at low temperature, which can understand multielectron tunneling to arbitrarily high order. In 2e case, the emission rate can be calculated by the convolution between $\rho \left(\hbar \omega \right)$ and $S_{\text{iet}}\left(\hbar \omega ,V_{b}\right)$ to the second order, which yields,

(4.4) $$\begin{array}{ll} U_{2e}\left(\hbar \omega ,V_{b}\right) & =G_{0}^{2}\rho \left(\hbar \omega \right)\int_{0}^{\hbar \omega }\rho \left(E\right)S_{\text{iet}}\\ & \;\times \left(E,V_{b}\right)S_{\text{iet}}\left(\hbar \omega -E,V_{b}\right)dE \end{array}$$

The 3e and higher order multielectron process can be similarly calculated by expanding the 2e case. Finally, the total emission spectrum is the summation over all contributing terms,

(4.5) $$U_{\text{total}}\left(\hbar \omega ,V_{b}\right)=\underset{i}{\sum }U_{ie}\left(\hbar \omega ,V_{b}\right)$$

The above model allows a normalization analysis method by dividing the measured spectra by a reference spectrum *U*_ref_ obtained at a significantly elevated bias *V*_ref_, which will result in abrupt slope change at *neV*_*b*_ position with *n* = 1, 2, 3…. Since the normalization removes the energy dependent plasmonic resonance and the reference spectrum can be treated solely as 1e photon emission given the high *V*_ref_ compared to other spectrum, each reduced spectra will only contain their respective ne (*n* > 1) emission variance, leading to the kink occurs at *ℏω* = *neV*_*b*_.

Fung and Venkataraman generalized this model to finite temperature by adjusting the limit in the calculation of the integral expression and adding a Heaviside function [166] (Figure 6(b)). Utilizing STM to repeatedly forming and breaking the atom contact between the tip and surface [167], they studied light emission and its dependence on the conductance based on a few thousands of junctions, yielding a super linear dependence of the overbias emission which is consistent with the multielectron process. The power law on the overbias emission further implies an electronic temperature on the order of 200 K, which rules out the blackbody-induced above-threshold emission which will be discussed in the next section.

### 4.3 Blackbody-like hot carrier above-threshold emission

To explain the observed above-threshold light emission, finite temperature effect and multielectron coherent tunneling have been proposed and described in Sections 4.1 and 4.2. Another mechanism that is drastically different from the above mechanisms involves the ultrahigh working temperature of the hot electron “gas” in the tunneling nanostructure, which could reach a few thousands of K. It was proposed that, when the tunneling current is sufficiently high, the electrically pumped charges cannot completely release their energy to the lattice before the next electron arrives. Under this scenario, rather than directly exciting a LSPR within the junction, those hot electrons can form a quasi-steady state distribution in the receiving electrode which can be described by an effective electronic temperature *T*_*e*_. Those hot electron distribution can radiatively emit a blackbody emission to the far field that energetically exceeds the electrical driven bias.

Downes et al. observed a blackbody-like light emission spectrum from a biased tunnel junction in 2002 [142], where they attributed the emission to the spontaneous photon emission from high temperatures electrons. Strong electronic heating is speculated through the weak electron–phonon interaction due to the strong suppression of bulk phonons in the nanoconstriction. When the characteristic size of the nanostructures is shorter than the length scales of electron-phonon coupling, the cooling mechanism through which the electrons lose their energy will mainly be limited to the electron collision with the interfaces and boundaries in the nanostructures. Fedorovich et al. developed a model for electron cooling inside a nanoparticle and related the electronic temperature in the nanoparticle to the injected electrical power by a formula [168],

(4.6) $${\left(k_{B}T_{e}\right)}^{2}-{\left(k_{B}T_{l}\right)}^{2}=\alpha IV_{b}$$

where *T*_*e*_ is the effective electronic temperature and *T*_*l*_ is the lattice temperature. *α* is a physical constant and is determined by the size of the nanoparticle, Fermi energy and the mass of the atom. When the effective electronic temperature is much higher than the lattice temperature, this formula is reduced to $k_{B}T_{e}∼\sqrt{\alpha IV_{b}}$ . Such temperature dependence is confirmed by Downes et al., giving an estimated electronic effective temperature around 1000–2000 K.

Later, Buret et al. revisited this idea and performed light emission measurements from nanofabricated planar electromigrated tunnel junctions [145] (Figure 6(c)). The giant above-threshold photon emission spectra at high conductance regime can be approximated by the blackbody-like hot carrier light emission model,

(4.7) $$U\left(\hbar \omega ,V_{b}\right)=\rho \left(\hbar \omega \right)\frac{\hbar \omega }{e^{\hbar \omega /k_{B}T_{e}}-1}$$

The effective electronic temperature dependence on the electrical condition (Eq. (4.2)) is also confirmed by investigating the power dependence between the photon intensity at a fixed wavelength and the electrical injected power on the logarithmic scale. They further measured the emission diagram through the conjugate Fourier plane, showing that the radiation pattern is governed by the symmetry of the nanogap. Similarly, Malinowski et al. examined the infrared light emission from nano hot electron gas from a biased gold atomic point contacts fabricated via mechanically controlled break junction [169]. The emission spectra in the infrared range (0.2 eV–1.2 eV) showed a good agreement with the emission of a hot electron gas with an effective temperature exceeding the melting point of the gold. Similar results have also been reported in planar tunnel junction made of epitaxial graphene on silicon carbide [170] (Figure 6(d)).

### 4.4 Above-threshold emission from plasmonic hot carrier recombination

The studies on above-threshold light emission since its first observation in 2002 [142] and the subsequent experimental and theoretical work have led to great insights, while consensus on the physical origin of this peculiar phenomenon has not been reached. Further systematic material studies based on different plasmonic structures are required to clarify the role of LSPR and the effect of different electrical conditions (current, conductance, etc.). In a series of studies by Cui and Zhu et al. [19], [92], [148], [171], [172], they have proposed a new physical mechanism that could potentially incorporate the contribution of both multielectron tunneling process and plasmonic hot carriers, depending on the current condition and the strength of plasmonic resonances in the tunnel junction structure, reconciling the conflict between previous experimental results and theories.

**Figure 7:**
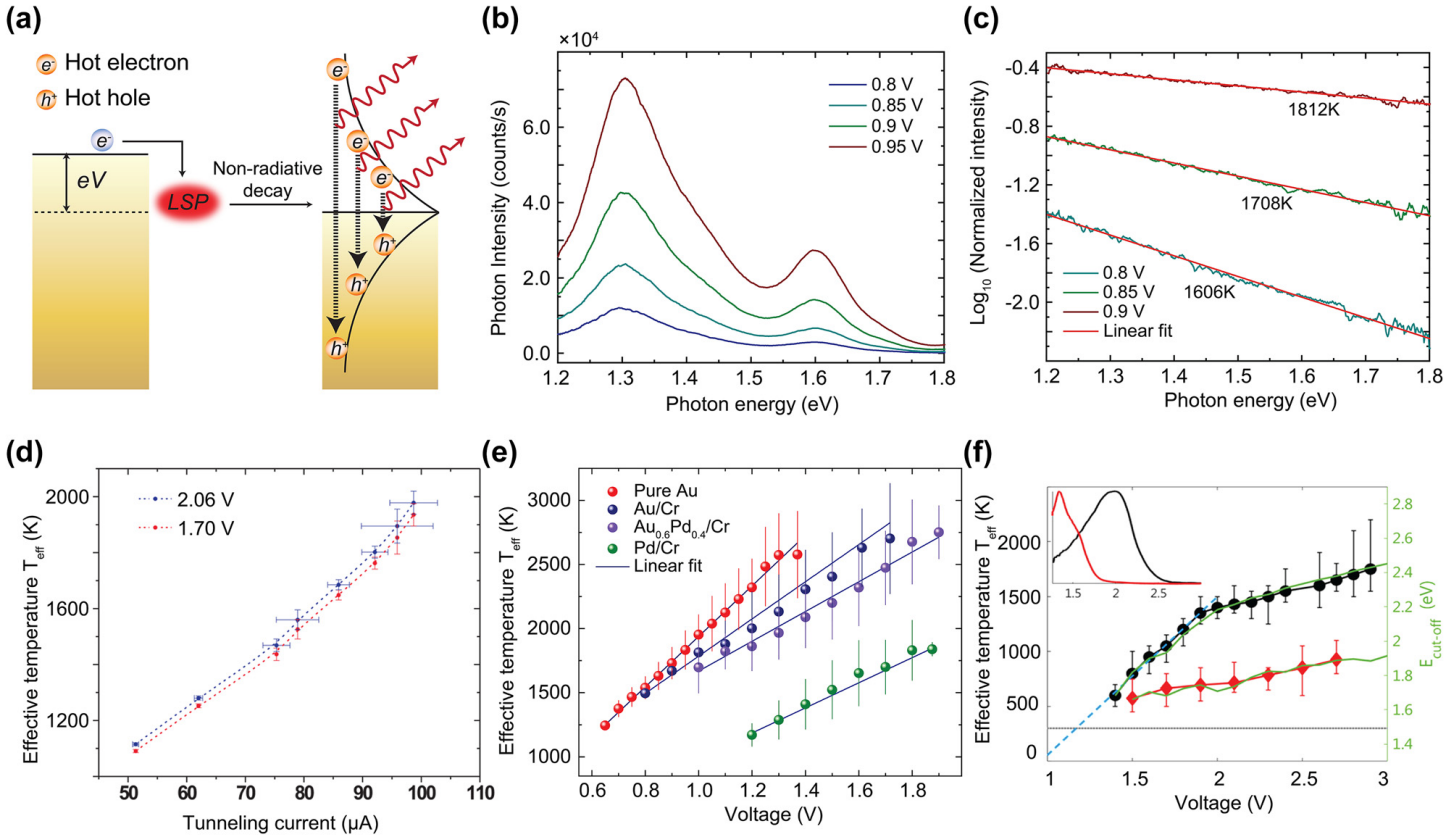
Experimental results for above-threshold light emission due to plasmonic hot carrier recombination. (a) Schematics of above-threshold light emission from plasmon induced hot carrier recombination [92]. (b) Measured light emission spectra under different biases from a planar Au electromigrated junction [92]. (c) Normalization analysis for the data in (b) to extract electron effective temperature through Boltzmann fitting [92]. (d)–(f) Extracted effective temperature from the light emission spectra versus the electrical condition (bias or current). (d) From a blackbody-like hot carrier induced overbias emission [145]. (e) From plasmon induced hot carrier light emission [92]. (f) Similar to (e) but from a vertical metal-semiconductor tunnel junction [174]. All figures are reproduced with permission from the American Chemical Society.

Above-threshold light emission involving plasmon-induced hot electron can be understand as follows: The LSPR excited by inelastic tunneling electrons first undergo a nonradiative damping process, which excites hot electrons and hot holes above and below the Fermi surface. Under the circumstances that the rate of tunneling electrons outpaces the hot carrier relaxation rate, a steady state hot carrier distribution can sustain, with its specific form determined by the time interval between successive electron tunneling and the hot carrier lifetime. Those hot carrier pairs will then radiatively decay through plasmon-mediated recombination, like recombination of electron–hole pairs in semiconductors, leading to a broadband photon emission which can readily exceed the voltage bias defined energy threshold (Figure 7(a)). Although the steady state hot carrier distribution is identical in functional form with the blackbody-like hot carrier mentioned above, this is reached by completely different mechanisms in terms of the associated dissipation process. In the previous case, electronic heating is realized through Joule heating by the injected charges, while in the latter scenario, heating is due to the nonradiative damping of the plasmons. Such plasmon mediated heating will exhibit a different dependence of the electronic effective temperature (Figure 7(b) and (c)), as discussed below.

When a plasmon with a quantized energy *ℏω*_*LSP*_ excited by electrical bias *V*_*b*_ is damped via Landau dephasing into the hot electron–hole pair, they are centered on Fermi level *E*_*F*_ and extends to *E*_*F*_ ± *eV*_*b*_, with a relaxation rate inversely proportional to the relative energy from the Fermi energy [36]. The effective temperature *T*_eff_, which corresponds to the average energy per carrier of the hot carriers in the steady state distribution, can be approximated as [92],

(4.8) $$k_{B}T_{e\mathrm{ff}}\propto \beta eV_{b}$$

where *β* is a material dependent parameter that relates to the plasmonic properties of the junction material. Another model which treats hot carriers formed within the electronic system as a viscous fluid and described the dynamics in the language of viscosity can yields a same linear voltage dependence of the effective temperature [173].

Such voltage dependence is experimentally confirmed by Cui et al. who statistically investigated the light emission from planar electromigrated tunnel junctions made from different plasmonic materials [92]. *T*_eff_ inferred from their normalization analysis show a linear relationship with the applied voltage, with less plasmonic loss material giving higher *β* (Figure 7(d–f)). The photon yield per electron differs by 4 orders of magnitude from pure Au to Pd/Cr junctions, suggesting that a plasmon-mediated heating mechanism is crucial in understanding the striking material dependence. Based on the same plasmonic hot carrier recombination model, Cui et al. [19] have also experimentally revealed that, under combined electro-optical excitation, the hot carrier effective temperature can rise drastically through the optically driven additional incoherent temperature *T*_0_ in the electrode, resulting in a giant increase (>1000×) in upconverted photon emission. Such hot electron based light emission junction demonstrates a broadband plasmonic switchable light source that is sensitive to the concurrent electrical and optical excitations.

Similar plasmon-mediated heating can be found in a vertical tunnel junction which has a metal–insulator–semiconductor structure [174]. A clear plasmon resonance is defined in such a structure through depositing a periodic array of gold bars with specifically designed length and width. Light emission can be seen from the structure when the electron inelastically tunnels through the barrier under bias. Effective electronic temperature *T*_eff_ inferred from the emission spectra scales linearly with voltage, suggesting the electron heating is realized through the nonradiative damping of plasmons.

**Figure 8:**
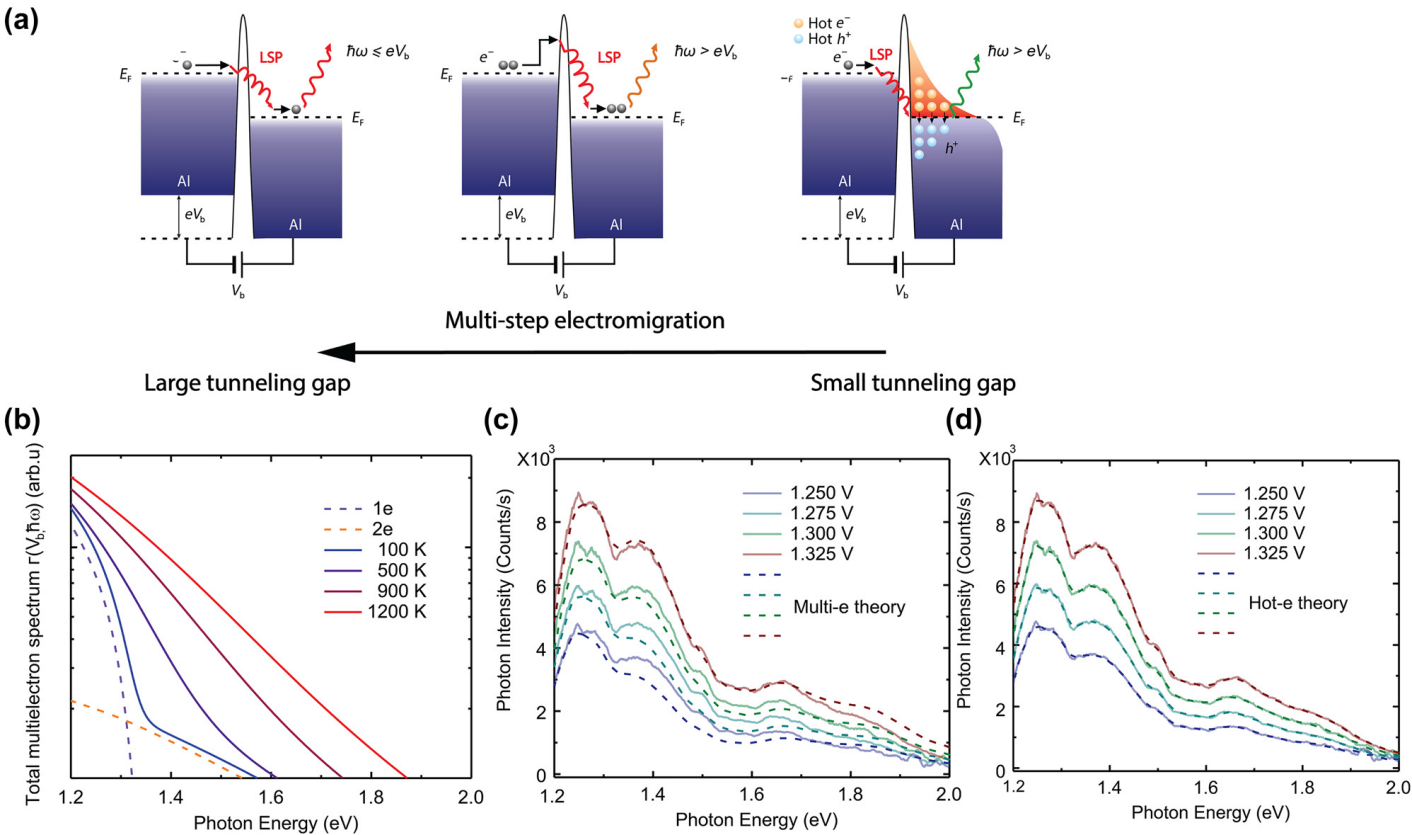
Observed emission mechanism crossover in Al tunneling junctions from multielectron tunneling to hot carrier recombination [171]. (a) Schematics for various light emission mechanisms and the how they evolve as the electromigration process continues. (b) Logarithmic plot of the total noise spectrum in the multielectron coherent tunneling model at different electronic temperatures. (c) Calculated spectra based on multielectron theory (dotted lines) plotted together with the experimental data. (d) Calculated spectra based on hot carrier theory, which shows a better match to the experimental data. All figures are reproduced with permission from the American Chemical Society.

More recently, Zhu et al. performed light emission measurements on planar electromigrated tunnel junctions made from pure Al, showing the crossover between the multielectron inelastic tunneling to plasmon-mediated hot carrier induced overbias photon emission when progressively altering the tunneling conductance (tunnel gap distance) [171] (Figure 8(a)). The good malleable property combined with a larger damping of the plasmon make Al stand out as a good candidate for probing different above-threshold mechanisms, as it is easier to controllably electromigrate an Al junction to larger resistances and the hot carrier generation channel can be potentially suppressed. Their experimental results (Figure 8(b–d)) suggest that hot-carrier-induced emission is most effective in the systems where there are strong plasmonic resonances and the electron interband scattering favoring nonradiative decay of local surface plasmons. This work could potentially solve the long-standing debate for the physical origin of above-threshold light emission.

### 4.5 Optoelectronic applications based on electrically driven plasmonic tunnel junctions

The below-threshold light emission associated with inelastic electron tunneling has intrinsically poor efficiency as only a minority of the tunneling electrons participate in the inelastic tunneling process.

**Figure 9:**
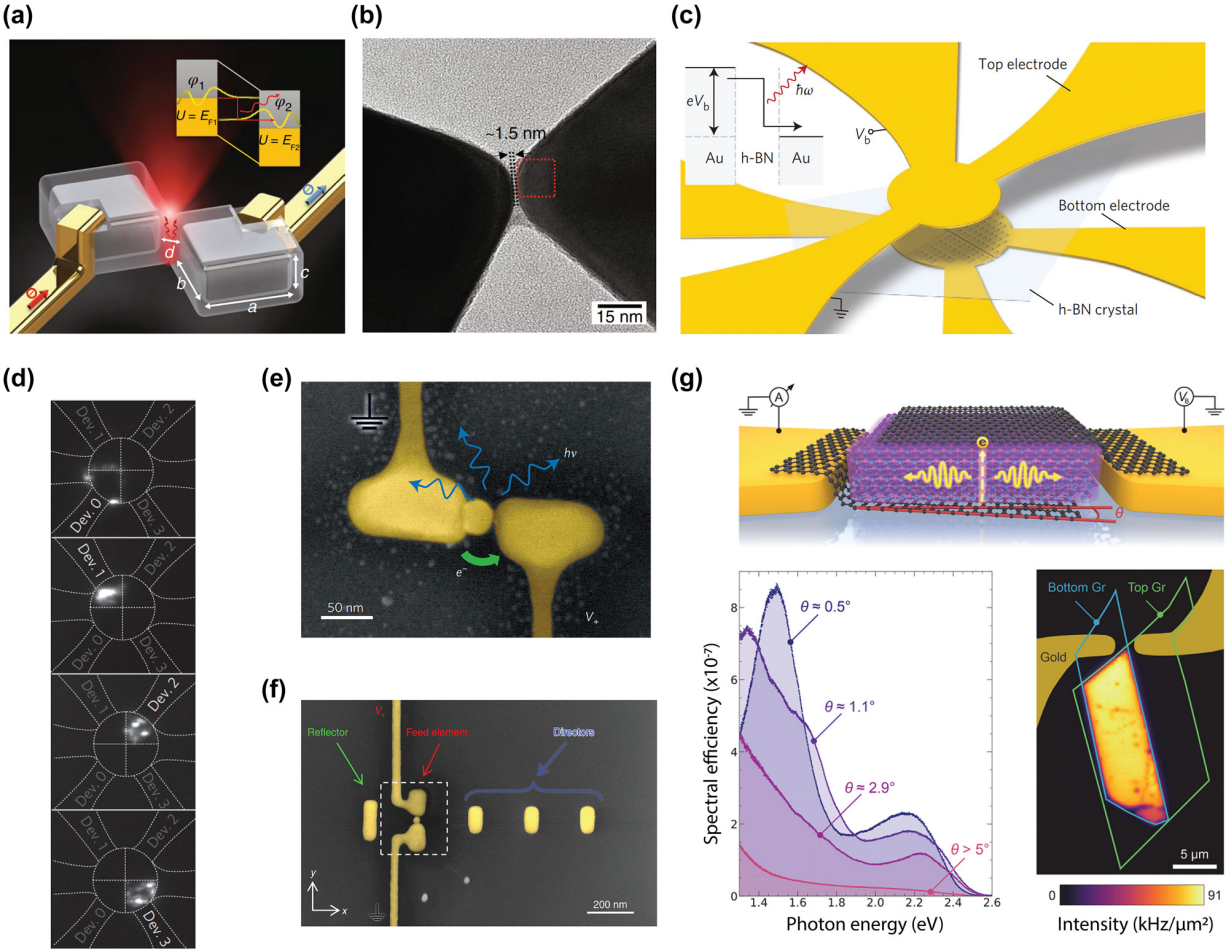
On-chip optoelectronic applications utilizing plasmon-enhanced light emission in molecular scale plasmonic nanostructures. (a) Diagram of a tunnel junction formed by two edge-to-edge Ag single-crystal cubes, with extended electrodes for electrical access [175]. (b) TEM image of the junction structure shown in (a). (c) A vertical tunnel junction structure formed by a few layers of hBN sandwiched between patterned Au electrodes [180]. (d) Images of four light emitting device shown in (c) at a constant applied bias 2.5 V. (e) SEM image of a lateral tunnel junction structure consisting of an electrically connected single-crystalline Au nanoantenna sandwiching an Au nanoparticle [177]. (f) SEM image of a Yagi–Uda antenna based on the junction shown in (e), which contains reflector, feed element and three directors [178]. (a)–(f) Are reproduced with permission from the Springer Nature Group. (g) Schematic of a twisted Gr/hBN/Gr tunnel junction for electroluminescence. Different spectra from various twisted angle are shown in the bottom left. A false color CCD image of the uniform light emitting area is shown in the bottom right [181]. Reproduced with permission from the American Chemical Society.

While below-threshold light emission induced by inelastic tunneling electrons can be understood by optical frequency current fluctuation mediated by the local plasmonic resonances, the efficiency of light emission is exceeding low, approximately one photon output per 10^4^–10^6^ electrons injected into the tunnel junction. Interest has been focused on improving the photon emission yield by engineering the plasmonic excitations of the metallic nanostructures. Qian et al. showed that enhanced light emission from inelastic tunneling can be achieved through geometry engineering and using single-crystalline material. After optimizing the local photonic density of states and the radiation efficiency, the light emission efficiency can reached up to 2 % at near-infrared regime [175], close to the theoretically predicted upper limit of 10 % [176]. In this setup, photons are emitted from an electronically biased junction created by two Ag nanocrystals (Figure 9(a) and (b)). Kern et al. fabricated in-plane antenna gap with a similar structure, showing that connecting to a precisely defined radiative antenna mode enhances light emission efficiency by two orders of magnitude and offers complete control over the characteristics of the emitted photons [177] (Figure 9(e)). Given the easy integration of the optical antenna and tunneling device, it becomes straightforward to introduce supplementary functionalities like gate electrodes, gap adjustments, and other passive or active optoelectronic elements. The same group has also further generalized their designs and demonstrated electrically-driven Yagi–Uda antennas featuring wavelength-scale footprints and very high directionality [178] (Figure 9(f)). This ensures the achievement of highly directional light emission via inelastic tunneling. Du et al. used Au/AlO*x*/Al junctions interconnected with plasmonic waveguides to create an electronic–plasmonic transducer [179], which allows efficient on-chip generation, manipulation, and readout of plasmons. Their junctions initiate plasmonic excitations within tunneling timescales, exhibiting spectral frequencies ranging from 300 to 350 THz.

Another advantage of optoelectronic devices based on inelastic electron tunneling is their inherent high speed. Unlike methods using intermediate excitations like electron–hole pairs, the response time of these devices is constrained solely by the electron tunneling time which is at femtosecond scale. Parzefall et al. fabricated a metal–insulator–metal tunnel junction with a vertical structure consisting of the top and bottom nanostructured Au electrodes, with exfoliated hBN in between as the insulator layer [180] (Figure 9(c) and (d)). Their device displays geometrically adjustable, polarization-sensitive resonances that can be modulated at frequencies up to 1 GHz. The same group had also used controlled stacking of graphene layers as a substitute for the top and bottom Au electrode [181] (Figure 9(g)). Photon emission occurs independent of the crystallographic alignment between the graphene electrodes. Devices with nearly aligned configurations display notable resonant attributes in both their optical and electrical characteristics, which diminish as twist angles deviate. These graphene/hBN/graphene tunnel junctions have the potential to serve as promising candidates for on-chip optoelectronics.

On-chip tunnel junctions have also opened the possibility of large-scale integration in array designs. By placing a single nanoparticle on the surface of a graphene tunnel junction, Namgung et al. shown that it is possible to electrically stimulate the plasmon of the nanoparticle without the need to provide individual electrical links to each nanoparticle [182]. This method enables the creation of plasmonic light sources using a scalable manufacturing process. The emission wavelength can be adjusted across a broad spectrum by tuning the nanoparticle and gap dimensions. A tunnel junction device with a similar structure based on a metal–dielectric–semiconductor heterostructure can be directly excited without additional optical components as a light source and shows the capability to detect the binding of small biomolecules such as antibodies [183]. Through the combination of internal field enhancement and an external matching grating, Göktaş et al. showed a 10-fold enhancement in internal efficiency and a 40-fold enhancement in overall quantum efficiency, leading to a novel class of solid-state light sources [184]. Kim et al. has also employed array designs of graphene nanogaps bridged by oxygen or nitrogen atoms, with the graphene nanogap formed through high-bias breakdown in a vacuum [185].

Qian et al. demonstrated that the introduction of a TiN/Al_2_O_3_ metallic quantum well (MQW) heterostructure enables the generation of resonant inelastic electrons tunneling, which could greatly enhance the efficiency of surface plasmon sources triggered through inelastic tunneling to close to unity [186]. This approach ushers in a new era of surface plasmon sources, not only enhancing the capabilities of high-performance plasmonic circuitry but also extending its utility to advanced optical sensing applications. In a similar vertically stacked tunnel junction structure, Zhang et al. shown that emission spectrum can be tuned by varying the width of the antenna. They showed an electron-to-photon conversion efficiency improvement exceeding three orders of magnitude when compared to a planar junction [187]. Wide-range wavelength tunability and broadband unidirectional emission are also achievable through a thiol molecule covered Ag nanowire (NW) cross-placed on a Au nanostrip [188] and a tunneling two-wire nanoantenna in the configuration of V shape on a transparent substrate [189].

Photon emission processes originating from electrically driven tunnel junctions stem from the interaction of stochastic fluctuations with the plasmonic modes of the surrounding environment. This interaction results in potential deviations from Poissonian statistics in the temporal correlations of the emitted photon sequence. Leon et al. performed time correlated photon counting measurements from a generic tip-surface metal junction [190]. When the junction is operated under a DC bias, individual tunneling events result in a sequence of visible plasmonic-origin photons, with a superbunching index 17 of this photon stream measured within the constraints of a 53-ps instrumental resolution. This discovery holds significant implications for quantum optics and cryptography, potentially leading to a robust design of on-chip entangled photon sources with atomic scale footprint. Malchow et al. had performed the a similar time correlation measurements on gold and silver nanostructures excited by laser pulses, showing signatures of photon bunching regardless of the geometry of the nano-object or the electronic band structure [191]. The correlation of photons released within a picosecond timeframe by nanoscale nonlinear sources of broadband radiation holds the potential for creating novel functionalities for temporal and spatial data processing.

## 5 Plexcitonic coupling mediated through hot carriers and electronically driven plasmonics

### 5.1 Strong coupling phenomena: classical, semi-classical, and full quantum model

A fundamental aspect of cavity quantum electrodynamics involves the interaction between cavity photons and quantum emitters [192]. The interaction strength that leads to this hybrid system is determined by the Rabi frequency, which describes the rate of energy exchange between quantum emitters and cavity photons. “Weak coupling” refers to the situation when the Rabi frequency is smaller than the decay rate of either the cavity or the quantum emitter, which has led to various applications primarily centered on enhancing the emission of quantum emitters [193], [194]. In contrast, strong coupling emerges when the Rabi frequency exceeds the decay rate of the combined average of the cavity and the quantum emitter. In this case, the energy levels of the combined system that govern emission will be modified accordingly and are eigenstates of a new hybrid system. They exhibit spectral Rabi splitting or correspondingly oscillations in time domain [195], [196]. Numerous applications have been achieved through strong-coupling, including precision measurement, quantum information transmission, as well as chemical and applications [197].

The classical description of a coupled system can be approximated using a harmonic oscillator model, which serves as an intuitive framework to understand various phenomena, such as electromagnetically induced transparency, level repulsion, non-adiabatic processes, and rapid adiabatic passage. In the simple scenario where two harmonic oscillators are coupled, such as two coupled pendula (oscillating at low frequencies) or a microwave field coupled with a resonating circuit, equations of motion can be solved via standard differential equations [198],

(5.1) $$x_{i}\left(t\right)=x_{i}^{0}\exp\left(-i\omega_{\pm }t\right)$$

with the new eigenfrequencies *ω*_±_ given by

(5.2) $$\omega_{\pm }^{2}=\frac{1}{2}\left[\omega_{A}^{2}+\omega_{B}^{2}\pm \sqrt{{\left(\omega_{A}^{2}-\omega_{B}^{2}\right)}^{2}+4\Gamma^{2}\omega_{A}\omega_{B}}\right]$$

where *ω*_*A*_ and *ω*_*B*_ are the eigenfrequencies for the two coupled harmonic oscillators, and Γ is the anti-crossing frequency splitting,

(5.3) $$\left[\omega_{+}-\omega_{-}\right]=\Gamma$$

Anti-crossing is the main characteristic of strong coupling and increases with coupling strength. The normal modes *ω*_+_ and *ω*_−_ do not pertain to the individual motion or position of either oscillator on its own. Instead, determining the normal modes requires analysing the temporal evolution of the motion of both oscillators in tandem. In other words, the normal modes represent hybrid modes resulting from the interaction of the two original oscillators. Consequently, relying solely on the description of the original oscillators is no longer sufficient; it’s imperative to employ hybrid modes when characterizing the system.

In the semi-classical models, the emitter is described as a quantum two-level system, akin to a spin-1/2 system, including an excited state and a ground state, each associated with their energies *E*_*e*_ and *E*_*g*_. The field inside the cavity remains classically electromagnetic. Here, we opt to present the results without diving into the derivation. Our intention is to convey fundamental physics, aspiring to make it comprehensible even to those without a background in quantum optics.

We first consider a single quantum emitter such as a dipole interacting with the field. The Hamiltonian describing the energy of the system is [199], [200]

(5.4) $$H=\frac{1}{2}E_{e}\left(I+\sigma_{z}\right)+\frac{1}{2}E_{g}\left(I-\sigma_{z}\right)+\hbar \Omega_{0}\left(\sigma_{+}+\sigma_{-}\right)\cos\left(\omega t\right)$$

where Ω_0_ represents the semiclassical Rabi frequency which is proportional to the dipole moment times the field amplitude. By performing the rotating wave approximation, the two matrix Hamiltonian can be diagonalized and has the eigenvalues

(5.5) $$E_{1}=-\frac{1}{2}\hbar \sqrt{\delta^{2}+\Omega_{0}^{2}},E_{2}=\frac{1}{2}\hbar \sqrt{\delta^{2}+\Omega_{0}^{2}}$$

where $\delta =\omega -\left(E_{e}-E_{g}\right)/\hbar$ and the generalized Rabi frequency is given by

(5.6) $$\Omega =\sqrt{\delta^{2}+\Omega_{0}^{2}}$$

The eigenstates of the system are effectively an even mixture of the ground and excited states, leading to Rabi oscillations that alternate between these two states. When at resonance *δ* = 0, the frequency of these oscillations coincides with the Rabi frequency.

In the quantum model, not only the quantum emitter is conceptualized as a two-level system, but the external field is also described as a lossy cavity that exhibits quantized fields. In the case of a single emitter, the Hamiltonian can be simplified using the rotating wave approximation [200],

(5.7) $$H=\frac{1}{2}\hbar \omega_{0}\sigma_{z}+\hbar \omega {\hat{a}}^{†}\hat{a}+\hbar \left(g\hat{a}\sigma_{+}+\text{h}\cdot \text{c}.\right)$$

*g* is proportional to the dipole moment, and $\hat{a}$ represents the annihilation operator for the quantized field, representing the removal of a photon. ${\hat{a}}^{†}$ corresponds to the creation of a photon. This is a general form of the Hamiltonian that describes the interaction between a quantized field and a two-level system. It is commonly referred to as the Jaynes–Cummings Hamiltonian. Diagonalizing the Hamiltonian will yield the following eigenvalues:

(5.8) $$E_{1n}=\hbar \left(n+\frac{1}{2}\right)\omega -\frac{1}{2}\sqrt{\delta^{2}+4g^{2}\left(n+1\right)}$$

(5.9) $$E_{2n}=\hbar \left(n+\frac{1}{2}\right)\omega +\frac{1}{2}\sqrt{\delta^{2}+4g^{2}\left(n+1\right)}$$

And the generalized Rabi frequency can be expressed as

(5.10) $$\Omega_{n}=\sqrt{\delta^{2}+4g^{2}\left(n+1\right)}$$

Here, a distinct disparity emerges when compared to the semiclassical case: a spectral splitting is evident, even when *n* = 0. This phenomenon is referred to as the vacuum Rabi splitting, and its presence is attributed to the electromagnetic vacuum fluctuations.

### 5.2 Optically driven plasmonic cavity coupled with quantum emitters

The introduction of plasmonic resonances into the context of strong coupling can be understood readily using the following picture: As light interacts with a metallic nanoparticle, it initiates oscillations among the free electrons at the same frequency as the field, resulting in the induction of a dipole moment within the particle. At resonance, denoted as *ω*_*c*_, the nanoparticle concentrates the electric field on its surface, amplifying its amplitude significantly beyond that of the incident field. This effect essentially transforms the particle into an optically driven “cavity”, with the quality factor *Q* defined as the ratio of the resonant frequency to the damping factor *Q* = *ω*_*c*_/*γ*. This factor dictates the extent of losses within the plasmonic cavity and its efficacy in storing plasmonic energy, which are critical for interactions with quantum emitters. Conventional plasmonic cavities tend to be very lossy and thus have low quality factors (significantly lower than those of dielectric cavities) due fast non-radiative damping in the metal and large linewidth of the plasmon resonance. However, recent years have witnessed great advances in the study of plasmonic systems at extreme atomic and molecular scale with pronounced strong coupling effects, which will be of great interest to summarize in this Section. We will provide a comprehensive survey of optically and electrically driven plasmonic cavities coupled with diverse quantum emitters. Also, we will examine the significance of photon or electrically excited hot carriers within the context of strong coupling.

#### 5.2.1 Plasmonic strong coupling with molecules

**Figure 10:**
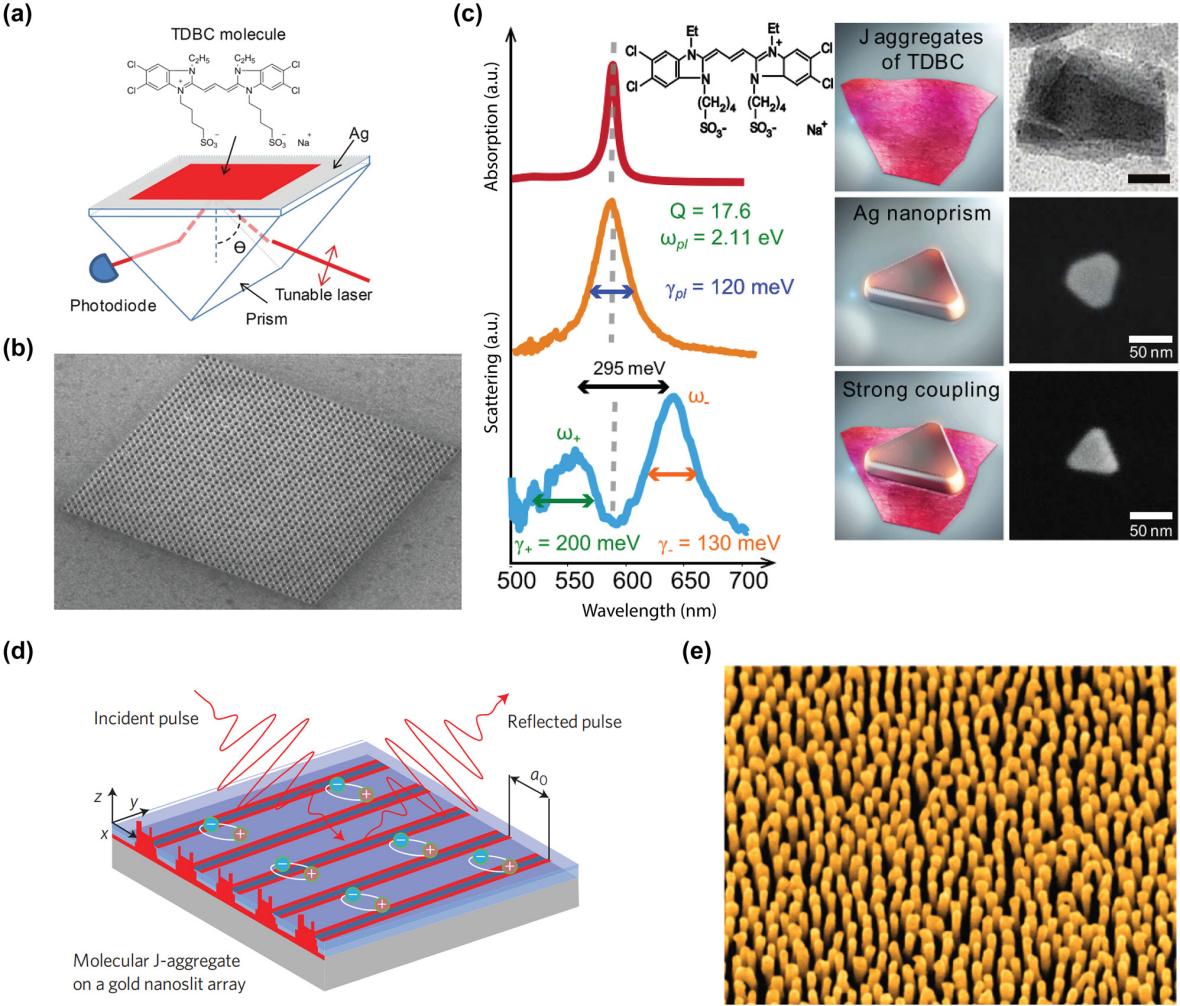
Experimental platforms for plexciton coupling. (a) Schematic representation of Kretschmann configuration, where the momentum of the incident light has been increased by the prism to obtain polaritonic dispersion curves [206]. (b) SEM image of hole array in a silver film fabricated through focus ion beam milling [207]. (c) The coupling between metallic nanoparticle plasmons and molecule excitons probed by extinction, scattering spectroscopy and electron microscopy. Spectra with various coupling strength are shown in the left [213]. (a)–(c) Are reproduced with permission from the American Physical Society. (d) A hybrid nanostructure consisting of a 50 nm thick film of J-aggregate molecules in a polymer matrix on a gold nano slit array for coherent ultrafast spectroscopy [208]. Reproduced with permission from the Springer Nature Group. (e) False colour SEM image of an ensemble of aligned Au nanorods supported by a transport substrate [210]. Reproduced with permission from the American Chemical Society.

J-aggregates represent a captivating category of fluorophore aggregates, emerging from the organized assembly of organic dyes. These aggregates boast intriguing optical characteristics, such as narrowly confined and red-shifted absorption and emission bands, as well as augmented absorption coefficients compared to the individual monomers. J-aggregates of organic dyes were originally identified by Scheibe and Jelley, both working independently in the 1930s. This discovery was based on the examination of the dye 1,1′-diethyl-2,2′-cyanine chloride [201], [202]. The initial observation of strong coupling between the excitonic absorption resonances of dye molecules and surface plasmon polaritons was made by Pockrand et al. [203]. He examined cyanine-based J-aggregated dye molecules deposited on a silver film. The dispersion of plasmon surface polaritons was studied by recording angular and wavelength scans in an attenuated total reflection configuration. Bellessa et al. performed the reflectometry experiments on the same platform, showing strong coupling between a surface plasmon and an exciton [204]. The Rabi splitting was observed at room temperature and reached a magnitude equivalent to the most substantial splitting in planar organic microcavities (∼180 meV). The emission from the low-energy plasmon–exciton mixed state was measured and displayed a significant shift compared to the emission from the uncoupled state. More recently, Symonds et al. performed a more comprehensive analysis of the strong coupling between surface plasmon and J-aggregates on planar metal surfaces [205]. Two distinct active materials were deposited onto a silver film: a cyanine dye J-aggregate and a two-dimensional layered perovskite-type semiconductor. They found that owing to the substantial interaction energies, calculations conducted at a constant angle can lead to an overestimation of the Rabi splitting by a factor of 2. Balci et al. had further shown that modulation of surface plasmon–exciton coupling can be achieved by regulating the damping of the plasmonic mode, a parameter determined by the thickness of the plasmonic thin film [206] (Figure 10(a)). The findings unveil the emergence of a plasmon–exciton hybrid state distinguished by an adjustable Rabi splitting, with energy levels spanning from 0 to 150 meV.

Up to this point, our emphasis has been on J-aggregate dye molecules coupled to a surface plasmon in a planar metal film, wherein prism coupling facilitates the coupling of incident light to the plasmon modes. In addition, surfaces with periodic modulation can also be employed, particularly when the period is comparable to the wavelength of light, enabling grating coupling. Dintinger et al. investigated the interaction between a J-aggregate and surface plasmon modes within a subwavelength hole array [207] (Figure 10(b)). The plasmonic structure is realized through focused ion beam milling on quartz coated with Ag film, with diverse hole periods ranging from 290 nm to 450 nm. Through the assessment of the impacts of the hole array period, angular dispersion, and J-aggregate concentration on the array’s transmission, strong coupling regime is reached, showing a substantial splitting of 250 meV. This splitting is induced by the high amplitude of the local field generated by the surface plasmons within the metallic structure. Utilizing the same technique, Vasa et al. fabricated periodic nanoslit arrays in a gold film, with period spacing ranging from 400 to 460 nm and spin coated a polymer film embedded with J-aggregated cyanine dye [208] (Figure 10(d)). In the J-aggregate/metal hybrid nanostructure, they observed ultrafast Rabi oscillations, signifying a coherent energy exchange between excitonic emitters and surface plasmon polariton fields. Their demonstration illustrates the coherent manipulation of coupling energy by exerting control over the exciton density at the 10 fs timescale.

In addition to the nanohole array or grating structures, alternative plasmonic nanostructures such as nanovoids and nanorods have also been studied. Sugawara et al. investigated hybrid emitting exciton–plasmonic composites created by coating arrays of spherical nanovoids embedded within a gold film with organic semiconducting molecular J-aggregate films [209]. The nanostructured gold is generated through a casting process involving electrochemical deposition through a template comprising self-assembled latex spheres with diameters of 600 nm. The resultant metallic mesh serves as the self-assembled template, offering a convenient means to manage the diameters of the pores and the uniformity of the array. Wurtz et al. presented their observation of strong coupling between J-aggregate and plasmon supported by an assembly of oriented gold nanorods [210] (Figure 10(e)). The gold nanorods are electrochemically grown within a substrate-supported porous template made of anodized aluminum oxide. Their results showed that the coupling can be readily engineered and is deterministic, as both the spatial and spectral overlap between the plasmonic structure and molecular aggregates are under control. Further, plasmonic nanostructures such as gold nanoshells [211] and arrays of metallic nanoparticles [212] can be used to achieve plasmon-exciton coupling with J-aggregates by adjusting their geometries. The larger splitting energy was attributed to the presence of a high concentration of molecules.

Zengin et al. realized strong light–matter coupling on the single-nanoparticle level [213] (Figure 10(c)). They demonstrated strong coupling between plasmons confined within a single silver nanoprism and excitons in molecular J-aggregates under ambient conditions. This highlighted that the combination of deep subwavelength mode volumes (*V*) and high-quality factors (*Q*) leading to $Q/\sqrt{V}∼6\times 10^{3}\mu m^{-3/2}$ , which is comparable to the performance of photonic crystal and micro-ring resonator cavities.

Plasmonic dimers are also recognized for creating regions of high field intensity known as “hot spots”. These hot spots can function as cavities capable of sustaining surface plasmon excitations and surpassing the diffraction limit by concentrating intense electromagnetic fields. Consequently, it becomes feasible to achieve strong coupling in plasmonic dimers with a reduced number of excitons positioned between the dimer elements. Schlather et al. studied the interactions between J-aggregate excitons and single plasmonic dimers, unveiling a novel strong coupling regime within individual plexcitonic nanostructures [214]. The plasmonic dimers were fabricated using planar techniques on an ITO-coated SiO_2_ wafer. Arrays of nanodisk dimers were defined through e-beam lithography. The plexciton dispersion curves exhibit anticrossings near the exciton transition energy, leading to substantial Rabi splitting from 230 to 400 meV.

While hot spots within plasmonic dimers can facilitate robust field enhancement, implementing top-down fabrication methods at the nanometer scale is challenging. In contrast, the bottom-up approach allows for finer control over the spacing between nanoparticles and metal films by applying insulating layers to them.

Like plasmonic dimers, another structure referred to as nanoparticle-on-mirror introduces a gap plasmon mode by positioning a gold nanoparticle on a gold film [215]. Quantum emitters are positioned within the gap situated between nanoparticles and the underlying metallic film. The gap distance, at a subnanometer scale, is controlled using molecular spacers. This approach is scalable, replicable, and can be readily characterized. Leveraging through this bottom-up nanoassembly, Chikkaraddy et al. shown that strong-coupling regime can be reached at room temperature and ambient conditions [28]. By reducing the cavity volume to below 40 nm^3^ and employing host-guest chemistry to arrange one to ten isolated methylene-blue molecules in a protective manner, they demonstrated that Rabi splitting of 300 meV for 10 methylene-blue molecules, gradually decreasing to 90 meV for individual molecules. Similar platform had been employed on J-aggregate molecules, exhibiting splitting of 377 meV [216].

In strong coupling, the combined average linewidths of the absorption/emission resonance and the optical/plasmonic mode should be narrower than the strength of the coupling. This condition is essential to characterize strong coupling phenomena. One might therefore expect that strong coupling is achievable primarily for molecules exhibiting a narrow absorption spectrum, like the above J-aggregates. Nonetheless, it’s noteworthy to mention that strong coupling has been observed even between surface plasmon polaritons and molecules characterized by a broader absorption spectrum. Pockrand et al. conducted experiments on squarylium and cyanine positioned on top of silver films, with an absorption line width around 80 meV [203], [217]. Reflectivity and dispersion curves for plasmon surface polaritons at the metal interface are measured, including both angular and wavelength scans. Hakala et al. reported strong coupling between surface-plasmon polaritons and Rhodamine 6G (R6G) molecules in sandwich structured samples with a 45 nm silver film and on the top a photo resist layer on four different concentrations of R6G [218], [219]. R6G has a linewidth of around 170 meV which is significantly larger than typical J-aggregates. Reflectometry measurements were conducted in the Kretschmann configuration, providing the dispersion relation of the energy associated with coupled modes in the system. The observed Rabi splitting was up to 230 meV and 110 meV.

#### 5.2.2 Coupling with 2D materials

2D semiconductors such as group-VI transition metal dichalcogenides (TMD) with the chemical formula MX2 (where M represents a transition metal and X is a chalcogen atom), exemplify robust interaction between light and matter. Compounds like MoS_2_, WSe_2_, among others, fall into this category, showing direct band gaps in the infrared and visible. These materials are promising candidates for applications in optics and optoelectronics [220]. Specifically, their substantial exciton binding energy and in-plane dipole moment can significantly amplify plexcitonic coupling [221], [222]. In contrast to other excitonic materials such as molecules or colloidal quantum dots, which have challenges in controlling their positions and orientations, TMDs offer several advantages due to their relatively uniform spatial distribution and precisely defined in-plane oriented transition dipoles, enabling robust exciton-light coupling under ambient conditions and at room temperature.

**Figure 11:**
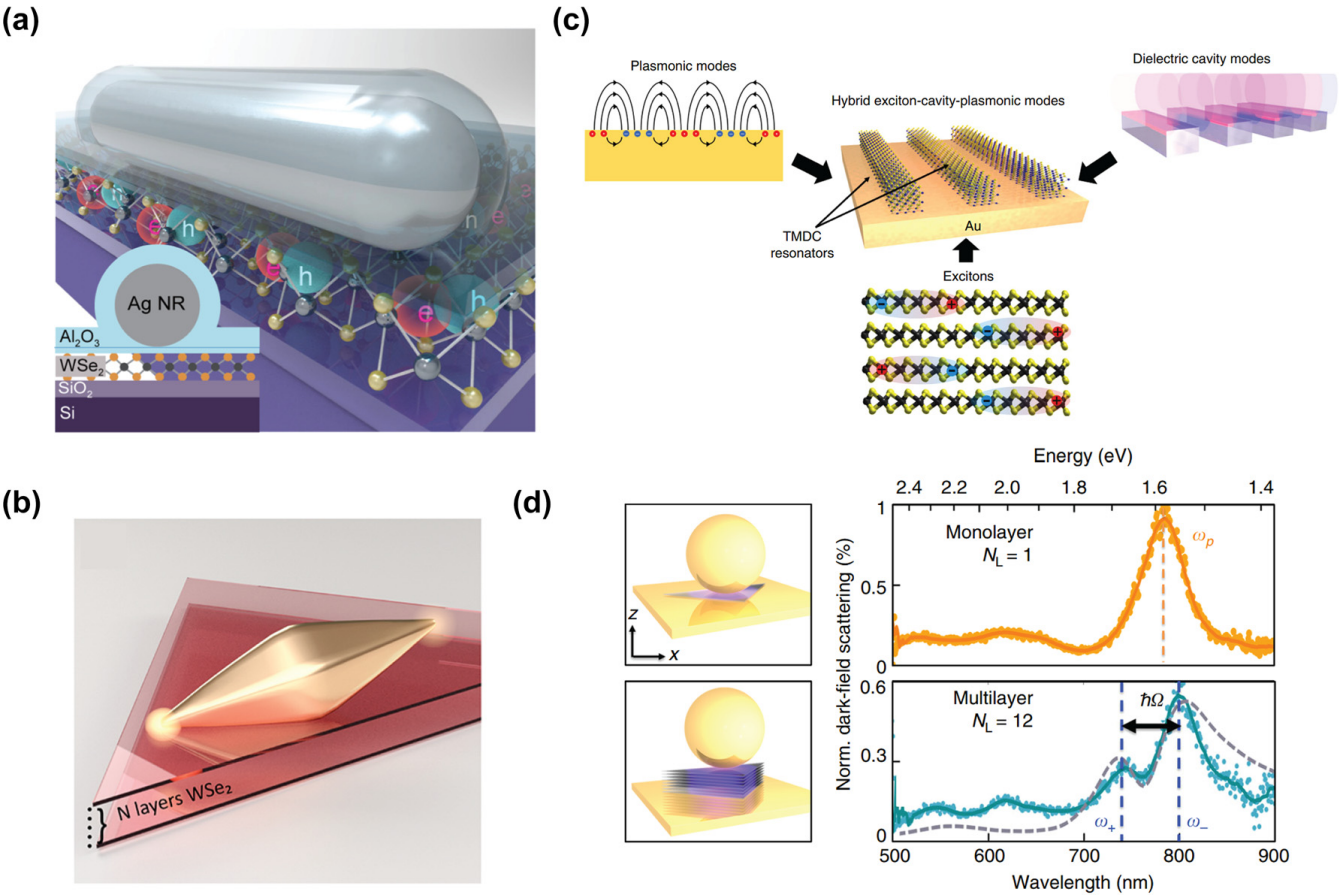
Plexciton coupling with TMD materials (a) A silver nanorod covered with Al_2_O_3_ on top of mono layer WSe_2_. Reproduced with permission from the American Physical Society. (b) A gold bi-pyramid placed on top of various thickness of WSe_2_ flake ranging from 1–8 layer [174]. Reproduced with permission from the American Chemical Society. (c) By combining specifically designed metal-dielectric nanostructures with engineered geometric dispersion and TMD resonators, a strong coupling photonic and electronic states can be achieved [175]. (d) A diagram describing the nanoparticle on mirror structure, where multilayer WSe_2_ is embedded inside the plasmonic nanocavities [223]. The dark field scattering spectra of individual structures on the right shows a single plasmon peak for monolayer WSe_2_ and mode splitting for multilayer WSe_2_ in the strong coupling regime [23]. (c)–(d) Are reproduced with permission from the Springer Nature Group.

Zheng et al. realized strong coupling between surface plasmons on an individual nanorod and excitons in a monolayer of tungsten diselenide (WSe_2_) [223] (Figure 11(a)). The plexciton system is created through a straightforward process of depositing and spinning a metal nanorod onto a monolayer of WSe_2_. This configuration resembles a nanoscale Fabry–Pérot cavity for the axisymmetric surface plasmon on 1D nanowire. The dispersion of plexcitons is evaluated by progressively redshifting the plasmon energy through the successive deposition of a dielectric layer (Al_2_O_3_) using ALD. Notably, the formation of plexcitons at room temperature reveals significant Rabi splitting, reaching up to 49.5 meV. Wen et al. utilized a similar setup involving a monolayer of WS_2_ and a single plasmonic gold nanorod, achieving Rabi splitting from 91 to 133 meV at ambient conditions [224]. Li et al. studied the nonlinear characteristics of these hybrid systems. This was accomplished by detecting second harmonic generation originating from a monolayer of WSe_2_ that was strongly coupled to a solitary gold nanorod [225].

Other plasmonic nanostructures like individual gold Bi-pyramids [226] (Figure 11(b)) or van der Waals semiconductor gratings [227] (Figure 11(c)) are also used to excite the hybrid light–matter states and their interference. In the later scenario, a multi-layer TMD is structured into nanoresonators on a template-stripped gold (Au) substrate. The grating period is deliberately tailored to achieve specific optical dispersion characteristics. They observed multipartite interaction and hybridization, resulting in robust coupling between three resonant excitations. This interaction exhibited an avoided crossing of 410 meV at room temperature. The nanoparticle-on-mirror nanocavities have also been used to explore the strong coupling with multilayer WSe_2_ [228]. Kleemann et al. argued that achieving strong coupling is unfeasible with monolayers due to the extensive exciton coherence size. However, they successfully resolved distinct anti-crossing patterns in devices with more than 7 layers, revealing Rabi splitting over 135 meV. Additionally, they illustrated how these structures enhance the potential for nonlinear exciton phenomena and presented evidence of superlinear light emission [23] (Figure 11(d)).

Beyond neutral excitons, transition metal dichalcogenides (TMDs) also accommodate charged excitons, facilitating dynamic adjustments of hybrid light–matter interactions via electrical means. Munkhbat et al. shown the capability to electrically modify charged exciton–plasmon polaritons within a hybrid system consisting of a monolayer of tungsten disulfide (WS_2_) and plasmonic nanoantennas [229]. By implementing electrical gating on monolayer WS_2_, it is possible to adjust the oscillator strengths of both neutral and charged excitons. This tunability extends beyond cryogenic temperatures to room temperature, under both vacuum and atmospheric conditions. This electrical manipulation facilitates a wide range of tuning from neutral to charged plexcitonic strong coupling.

Furthermore, by placing a gold nanoantenna on top of the MgF_2_ substrate, mode-selective coupling of vibrational resonances can be realized through a metal scanning probe tip, enabling intramolecular vibrational energy transfer [230]. Time-resolved infrared vibrational nanospectroscopy can thus determine Purcell-enhanced vibrational lifetimes of molecular vibrations, accomplished by tuning the IR nanoantenna across coupled vibrations. Similar spectroscopy, either in a vertical [231], [232] or a planar geometry [172], [233], can potentially pave the way for creating, investigating, and manipulating single-emitter plasmon hybrid quantum states, offering opportunities for a wide range of applications spanning from optoelectronics to quantum information science, even at room temperature.

#### 5.2.3 Coupling with semiconductor nanocrystals

Another promising avenue to leverage high intensity excitations in strong coupling is the utilization of quantum dots, commonly referred to as semiconductor nanocrystals. Also referred to as “artificial atoms,” quantum dots are small particles of a few nanometers which are synthesized in solution using colloidal chemistry and have remarkable optical and electronic properties [234]. Srinivasan et al. employed a fiber taper waveguide to directly conduct optical spectroscopy on a system comprising a quantum dot integrated within a microdisk [196]. Optical fiber is coupled to the system through a piezo-actuated stage, directing the cavity’s reflected and transmitted signals to photodetectors and a spectrometer. This approach also enables them to investigate the steady-state nonlinear characteristics of the system, revealing a saturation of the cavity-quantum dot response with fewer than one intracavity photon. Englund et al. performed measurements on a InAs quantum dot coupled to a GaAs photonic crystal cavity, showing the quantum dot significantly alters the transmission and reflection spectra of the cavity [235]. They further observe the phenomenon that tuning the quantum dot energy across the cavity leads to variations in the coupling strength between the two entities. Similar experiments that enabled the observation of both weak [236] and strong coupling [195], [237] regimes of interaction between a photonic crystal cavity and a single quantum dot in photoluminescence have also been reported.

### 5.3 Electrically driven plasmonic cavity coupled with quantum emitters

Accessing individual strongly coupled emitters has predominantly relied on far-field optical probes with spatially limited resolutions [23], [238], [239], [240]. This approach hinders efforts towards miniaturization of strongly coupled systems and large-scale integration. In this light, electrically driven plasmonic nanostructures hold the potential to serve as a viable method to achieve strong coupling phenomena at the fundamental atomic and single molecule limit. By positioning TMDs or molecules supporting excitons near those electrically driven plasmonic nanostructures, it is feasible to access the extreme near field of the LSPR for strong coupling observations. Near-field probes offer a promising approach to characterize polaritonic physics. This is particularly useful since far-field optical methods may inevitably capture extra bright emissions arising from uncoupled excitons, obscuring insights into the strong coupling phenomenon of coupled excitons.

#### 5.3.1 Single molecule electroluminescence

Single-photon sources are pivotal in quantum information and computing technologies. Electrically driven single-molecule light emitters stand out as one of the promising candidates to create efficient single-photon sources. The STM offers the capability to selectively stimulate molecular electroluminescence through inelastically tunneling electrons. It also enables the enhancement of emission through the localized fields generated by nano cavity plasmons. By carefully optimizing the molecule, spacer, STM tip, and substrate, the emission intensity can be sufficiently strong and stable, which allows the measurements of second-order photon correlation. The observation of photon antibunching effect serves as compelling evidence for the single-photon emission characteristics in single-molecule electroluminescence [241]. In addition, individual molecules can exhibit coherent coupling with a plasmonic nanocavity, leading to the emergence of Fano resonances induced by interference. These discoveries pave the way for novel approaches in fabricating electrically driven quantum emitters, as well as for exploring intermolecular energy transfer, field-matter interaction, and molecular optoelectronics, all at the level of single molecules [231].

**Figure 12:**
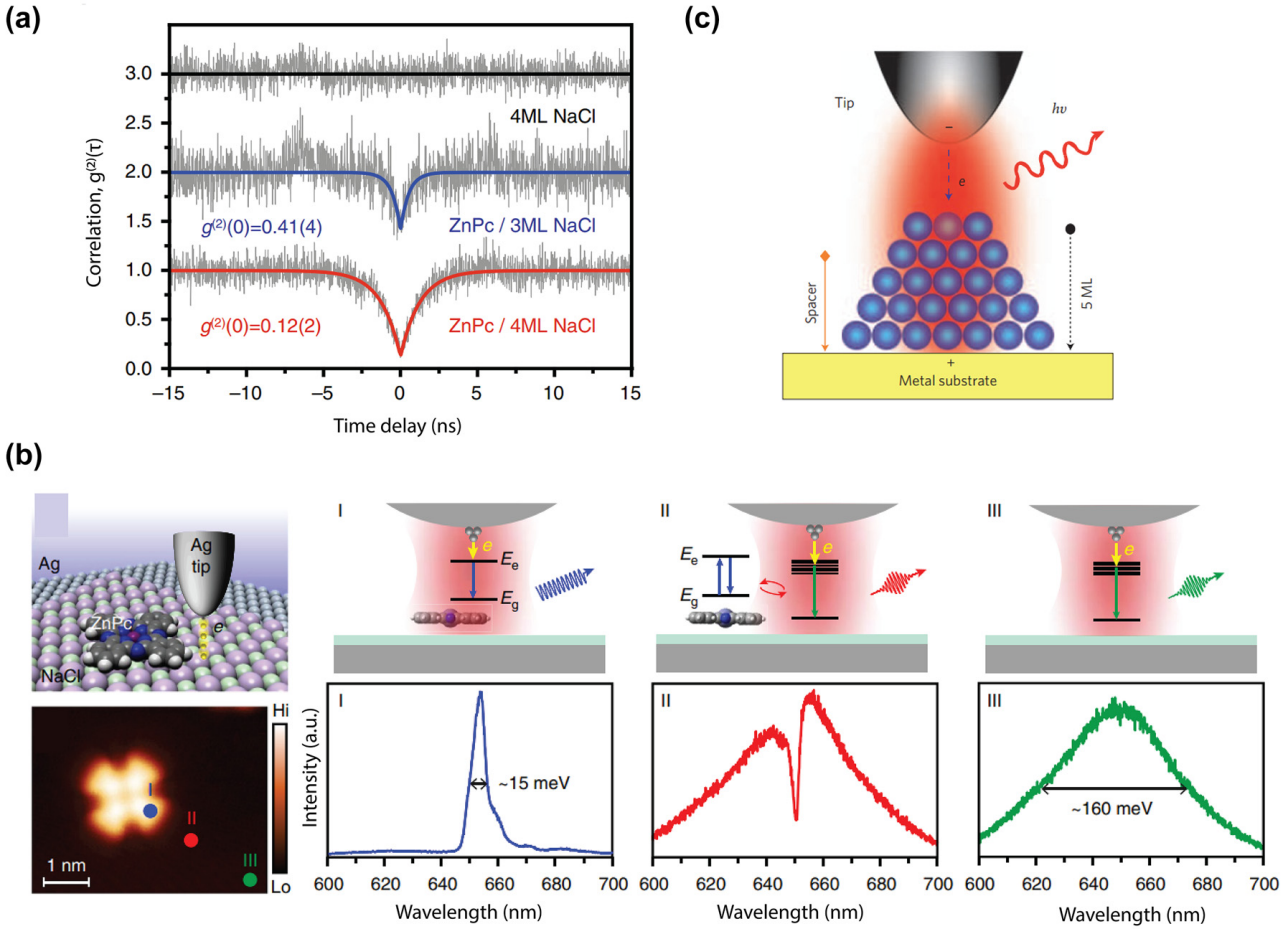
Experiments of single molecule electroluminescence. (a) Second order correlation measurements showing the antibunching feature of single molecule electroluminescence at ZnPc-lobe [244]. (b) When a tip scan through different positions of a molecule, different types of electroluminescence can be observed. A typical Fano line shape can be explained by the interaction between the nanocavity plasmon and the two-level emitter in proximity [246]. (c) A junction geometry with multilayer molecule stacking and localized electrical nanotip excitation [252]. All figures are reproduced with permission from the Springer Nature Group.

Zhang et al. observed electrically driven single-photon emission from a precisely positioned individual molecule situated within a meticulously controlled nanocavity between a STM tip and the substrate [241]. Their configuration involves an isolated ZnPc molecular emitter electronically separated from the Ag (100) substrate by a thin layer of sodium chloride (NaCl). The detection of antibunching dips indicates single-photon emission behavior with measured *g* ^(2)^(0) value down to 0.09. They also demonstrated the potential to create arrays of single-photon emitters, showing the promise of scalable quantum nano-optoelectronics. Antibunching has also been observed for organic dye molecule dibenzanthanthrene [242], vacancy center in diamond [243] and C_60_ molecules [244] (Figure 12(a)). The fluctuation behavior has also been studied to understand the dynamics of the single molecule electroluminescence [245].

When a quantum emitter resonant with nanocavity plasmons, quantum interference occurs due to the coherent interaction between the emitter’s discrete state and the nanocavity plasmons’ continuum-like state, leading to Fano resonance. Single-molecule Fano resonance have been realized via the excitation of tunneling electrons when an STM tip is brought into close proximity to an isolated ZnPc molecule [246] (Figure 12(b)). The coupling strength between the individual molecule and the plasmonic nanocavity can be adjusted by manipulating the separation distance between them. This coupling evolves from displaying a Fano lineshape to a broad plasmon profile as the tip is displaced away from the molecule. Various energy detunings are also demonstrated by altering the shape of the STM tip. This modification led to tunable plasmonic resonance from 620 to 690 nm. Other studies on phthalocyanine molecule [247] and gold nano particle [248] coupling to the plasmonic nano cavity yields similar Fano resonance spectral shape.

In principle, molecular fluorescence can be manipulated across a broad energy spectrum, and specific frequencies can be chosen by adjusting the emission through resonance enhancement near plasmonic structures. This allows for precise control of the emission from different vibronic transitions. Due to substantial fluorescence quenching when molecules are directly adsorbed onto metal surfaces [249], these investigations centered around molecules that were isolated from the metal substrate through the presence of insulator layers or molecular multilayers [250], [251]. Electrical excitation via STM offers an added advantage in that it can dynamically adjust the spectral profile of nanocavity plasmons on the spot by manipulating the tip’s state. Subsequently, the resonance mode can be directly measured. Furthermore, the utilization of tunneling electrons for excitation, which is non-optical in nature, eliminates potential complexities arising from incident light disturbance (such as Rayleigh scattering). STM not only offers the essential spatial resolution to discern the adsorption arrangement of an individual molecule on a surface, but it also affords the capability to finely manipulate the local environment around the molecule with atomic-scale precision. Molecular electronic excitations and radiative decay offer opportunities for tunneling transport devices where tunneling carriers can activate molecular function, such as in the case of sensors and organic light-emitting diodes (OLED).

Dong et al. presented the occurrence of resonant hot electroluminescence, originating directly from higher vibronic levels of the singlet excited state within porphyrin molecules confined within a nanocavity of a scanning tunneling microscope [252] (Figure 12(c)). This effect is achieved by fine-tuning the frequency of plasmons to match the spectral requirements. These observations unveil novel optoelectronic phenomena that hold utility in investigating the near-field properties of nanocavity plasmons and their corresponding field enhancement. This characteristic allows for the active manipulation of radiative pathways of molecular emitters through the substantial enhancement of resonance in both excitation and emission processes.

By employing scanning tunneling microscopy and presenting a comprehensive investigation into the electroluminescence exhibited by thin C_60_ films on Ag(111) and Au(111) surfaces, Große et al. shown the mapping capability of the electroluminescence and demonstrated it is possible to definitively distinguish between the inherent radiative recombination of electron–hole pairs within the organic film and the emission resulting from plasmons induced by the tip [253]. Reecht et al. reported electroluminescence exhibited by a polythiophene wire positioned between a metallic surface and the tip of a scanning tunnelling microscope, showing that the spectral and voltage variations of the emitted light align with the fluorescence pattern of the wire junction under positive bias and notably suppressed when the polarity is reversed [254]. The emission mechanism and polarity dependence closely resemble the behavior observed in organic light-emitting diodes. Similar unipolar behavior of the electroluminescence observed in the engineered tripodal molecule indicates that the molecule is probably stimulated by hot electron in injection, leading to the subsequent radiative decay of the excited state [255]. These findings hold the potential to open new avenues for the creation of nanoscale OLEDs.

Svatek et al. demonstrated the integration of organized monolayers of organic molecules, stabilized through hydrogen bonding, onto the surface of exfoliated few-layer hBN flakes [256]. These systems can be integrated into van der Waals heterostructures, resulting in the formation of a hybrid tunneling diode that merges molecular and two-dimensional elements. Molecular electroluminescence shows that tunneling electrons stimulate embedded molecules into singlet states using a two-step mechanism involving an intermediate triplet state through inelastic scattering, provide a new route for the on-chip spin-based applications of triplet generation. Yonemoto et al. described the creation of a nanogap light-emitting electrochemical cell (LEC), which involves the deposition of a light-emitting polymer and an ionic liquid onto a gold nanogap electrode [257]. The device exhibited a substantial electroluminescence intensity at a wavelength of 540 nm, aligning with the emission peak of the polymer. This establishes that the nano-LEC concept holds promise for realizing current-induced light sources at the molecular scale. Du et al. reported on-chip molecular electronic plasmon sources which utilize tunnel junctions composed of self-assembled monolayers positioned between two metallic electrodes [258], [259]. This setup demonstrates the capacity to control plasmon intensity through alterations in the chemical structure of the molecules. Additionally, they achieve bias-selective excitation of plasmons using molecular diodes.

#### 5.3.2 Electrically driven strong coupling with TMD

More recently, TMDs have also been used as a potential platform to realize plexciton strong coupling based on electrically driven approach. Papadopoulos et al. explored a new way to electrically generate excitons in WSe_2_ by placing the 2D material on top of a vertically stacked gold-hBN-graphene tunnel junction [260]. The TMD and tunnel junction are intentionally kept electrically isolated, effectively preventing the generation of excitons through direct electron–hole injection. The measured emission spectrum exhibits a notable peak at the exciton energy. This phenomenon is attributed to the generation of excitons through energy transfer from tunneling electrons mediated by dipole–dipole coupling. They concluded that a significant enhancement of non-radiative modes is presented within the local density of states at the exciton energy, which plays a pivotal role in facilitating efficient energy transfer from the tunnel junction to the TMD.

In contrast, Zhu et al. performed electroluminescence measurements on the TMD-junction with a different structure, where WSe_2_ is directly transferred on top of the plasmonic metallic tunnel junction [24]. The ultra-narrow gap spanning sub-nanometer dimensions between the metallic electrodes functions as an exceptionally confined plasmonic nanocavity. Within this nanocavity, incoherent photons are generated through the recombination of hot carriers. The introduction of the TMD near the nanogap’s localized surface plasmons leads to the emergence of plexciton polaritons. These polaritons exert a pronounced in fluence on the radiative local density of states, which in turn governs the emission of light originating from the electrically driven hot carriers. Rabi splitting exceeding 50 meV indicating the electrically driven TMD-junction system enters the strong coupling regime, both for unpolarized and polarization dependent emission. In this context, electroluminescence serves as a novel near-field probing technique, providing access to exceptionally localized information. The formation of plexcitons in turn fine-tunes the recombination process of hot electrons and holes within the metal, which can be intentionally designed to manipulate the energetic relaxation of hot carriers within metals.

## 6 Other applications and future research directions

Recent years have seen a rapid increase in research on other applications of plasmon-induced hot carriers and electrically driven processes in plasmonic structures. Plasmon-enhanced photodetection and photocatalysis are two prominent examples. Here we will discuss important recent advances on these topics and highlight some ongoing debates, particularly around the physical origin of the plasmon-enhanced photocatalytic effects. In the end, we will provide our views on potential future directions and challenges.

### 6.1 Hot carrier photovoltaics and photodetection

Plasmon-induced hot carriers hold significant importance in light harvesting, as they possess the capability to mitigate the constraints associated with semiconductor-based devices. Under illustration, plasmonic hot carriers can harness near-infrared light, characterized by photon energy lower than the bandgap of semiconductors. Furthermore, they enable the utilization of selective wavelength or polarization modes with narrow bandwidth. A critical issue that hinders the practical applications of photovoltaics is the low absorption of plasmonic metals and rapid hot carrier thermalization. Hence, it becomes imperative to devise strategies that enhance the rate of hot carrier generation while simultaneously minimizing hot carrier losses.

Kang et al. shown that the ordered structure of metal-semiconductor plasmonic nanodiodes can engineer the hot carrier flow and thus boost the hot carrier generation through properly geometrical modification of the plasmonic nanostructures [261]. A comparable plasmonic nanohole electrode design combined with an organic Schottky barrier photodetector is shown to have the capability to drastically improve the photon emission rate through engineering the localized surface plasmon resonance, which could help the hot carrier harvesting [262]. Other Inorganic/organic semiconductor nanocrystals materials consisting of colloidal halide perovskite [263], quantum dots [264], [265] and graphene [266] have also been proposed to facilitate hot carrier generation and subsequent collection. Furthermore, it is possible to tune the wavelength dependent response of the photodetector based on those semiconductor structures by controlling the degree of hot carrier injection, reaching a response up to long wavelength infrared of 55 um [267].

Other than the complicated semiconductor plasmonic nano devices for potential hot carrier photovoltaic applications, simple plasmonic metallic tunnel junctions have also shown the ability to boost hot carrier generation and subsequent transport. Zolotavin et al. shown that single-metal Au nanowires and nanowires with nanogaps formed by electromigration can exhibit large open-circuit photovoltages up to tens of mV and a sharp polarization dependence under optical illumination [268]. The hot carrier generation rate can be further enhanced by removing the adhesion layer and changing the width of the nanowire [19]. Such simple structure for hot carrier generation also demonstrates the potential application of detecting lattice distortions [269], identifying impurities [270], functioning as photodetectors [271], and serving as a directional hot carrier generator [272].

### 6.2 Plasmon-enhanced photocatalysis and debates on the physical mechanism

Plasmon-enhance photocatalysis concerns the significantly improved chemical reaction rates and product yield due to the use of plasmonic nanostructures. However, there are still open questions and debates on the exact role and quantitative contribution of hot carriers and other plasmonic processes such as localized heating. On one side, one potential physical mechanism of hot carrier involved photocatalysis is the significantly higher energies carried by optically and electrically excited hot carriers, surpassing those arising from thermal excitations. Hot carriers can migrate into unoccupied energy levels of acceptor molecules nearby a plasmonic nanostructure, triggering various chemical processes. It should be noted that these capabilities can be potentially limited by the rapid relaxation dynamics through which all the excess energy dissipates into heat. On the other hand, the strong confinement of thermal processes naturally associated with plasmonic nanostructures, and their large absorption cross section can produce highly localized lattice heating which can enhance the chemical reactions and has been leveraged in controllable photothermal applications.

The high absorption cross-section by plasmon-resonant noble metal nanoparticles held particular interest for altering the optical characteristics of metal oxides [273] and enhancing the photochemistry of aborbate molecules at the surface of nanopoarticles [274], [275]. A simple and straightforward reaction catalyzed by hot electrons involves the room-temperature dissociation of H_2_ at the surfaces of Au nanoparticles [276], [277], where the hot electrons possess the capability to transfer into a Feshbach resonance of a hydrogen molecule adsorbed onto the surface of the Au nanoparticle in the transient state. They explored this process by discerning the creation of HD molecules stemming from the dissociation of H_2_ and D_2_, while also examining how the rate of HD formation is influenced by factors such as the size of Au nanoparticles and the wavelength of incident light. Similar plasmonic Au, Al nanostructures had been used as photocatalyst for highly efficient direct decomposition of HS_2_ [278], selective photo detoxification of sulfur mustard simulant [279], solvated electron generation in solution [280] and hydrogen evolution [281], [282]. The hot-electron-driven partial oxidation of ethylene using O_2_ to produce ethylene oxide is also investigated to study the plasmon-driven photodissociation of O_2_ [29], [283], [284], where the photocatalyst was composed of Ag nanocubes, exposing Ag(100) facets to ensure heightened reactivity and a precisely defined plasmon resonance.

Antenna-reactor complexes have recently been developed, such as a plasmonic nanoparticle (the antenna) adorned with reactor particles, including islands, clusters, or individual platinum group metal atoms [285], [286]. An antenna-reactor based on Cu–Ru complex [31] had been utilized as a plasmonic photocatalyst to efficiently reduce the thermal activation barrier for ammonia decomposition due to the Ru serving as the ideal binding site for nitrogen intermediate species during the reaction [287]. While the inclusion of platinum group metal reactors significantly enhances photocatalytic performance, their limited availability and high cost have led the researchers to substitute them with more readily available transition metals, which is especially crucial for industrial-scale reactions like ammonia decomposition [288], [289]. The earth-abundant metal Fe, however, is far less reactive compared to Ru as a plasmonic photo catalyst since the strength of Fe–N bond is too strong to facilitate the desorb of the product [290]. In this regards, Yuan et al. prepared Cu–Fe and Cu–Ru antenna reactors through coprecipitation, showing Fe active sites in a Cu–Fe complex structure can have comparable efficiencies with Ru for ammonia decomposition when illuminated with ultra-fast laser pulse [291]. Lin et al. investigated planar Al nanodisk antenna combined with spatially separated Pd and Fe nanodisks for H_2_ dissociation and NH_3_ activation, demonstrating that precisely engineered antennas combined with multiple reactors can progressively control the chemical reaction ranging from simple to complex [292].

Different views were proposed to explain these recent plasmonic photocatalysis results from purely (lattice) thermal Arrhenius activation [293], [294], [295], [296] rather than the hot carrier-mediated chemical conversion. The exponential sensitivity of the reaction rate suggests that an underestimate of the true temperature in the reaction environment is possible, therefore yielding an underestimate of the thermocatalysis reaction rate by three orders of magnitude. Zhang et al. introduced a method through illuminated rhodium nanoparticles on oxide supports to catalyze CO_2_ methanation reaction to distinguish the thermal and nonthermal contributions from plasmon-enhanced catalysts. Effective thermal and nonthermal reaction rates under illumination can be extracted via the simultaneous measurements of overall reaction rate and the top- and bottom-surface catalyst bed temperature, revealing a synergistic behavior for both thermal and nonthermal(plasmon-enhanced) catalysis [297]. To further disentangle these thermal and nonthermal effects, Li et al. applied a novel indirect illumination technique as well as the precise monitoring of the thermal profile to the same CO_2_ methanation, showing a clear signature of nonthermal effects in the Rh/TiO_2_ plasmonic photocatalyst [298]. However, those control experiments inevitably omit the spatial temperature variances which can significantly impact local catalytic rates, as thermal camera technique typically provide only average temperature information [293]. Clearly, it is desired to have the capability to measure the nanoscale temperature distribution through thermometry methods [299], to distinguish between lattice temperatures and possible effective electronic temperatures, and to design critical control experiments to reproduce the nonuniform temperature distribution [295], [296]. Further discussions regarding the debates can be find in other review [300].

Beyond optically excited plasmonic catalysis, electrically driven plasmonic nanostructures can also facilitate chemical reactions. Wang et al. demonstrated that the generation of hot electrons renders nanoscale tunnel junctions exceptionally reactive, enabling the occurrence of tightly confined chemical reactions that, in turn, have the capacity to influence and modulate the tunnelling processes [301]. An array of electrically driven plasmonic nanorods, incorporating as many as 10^11^ tunnel junctions per square centimeter are fabricated, showing the activation of oxidation and reduction reactions within the junctions via hot electrons, triggered by the presence of O_2_ and H_2_. The same group has developed an artificial synapse that exhibits both electrical and optical memory effects through chemical transformations occurring within plasmonic tunnel junctions [302]. When subjected to bias, electrons that tunnel into plasmonic nanorods under a low voltage are harnessed to encode information into the tunnel junctions through chemical reactions mediated by hot electrons interacting with the environment. Alternatively, in an optical configuration, external illumination can be utilized to write information by exciting hot electrons within the plasmonic nanorods. The described structure holds the potential for application as multilevel non-volatile memory, logic units, or artificial synapses in forthcoming electronic, optoelectronic, and artificial neural network systems. Here we note that the physical mechanism of hot carrier enhanced photocatalysis has inspires some debates [296], [303], [304], [305], particularly around the relative contribution of thermal and non-thermal hot carriers in promoting catalytic performance.

While the plasmon-enhanced catalytic effects have been observed in many studies, it can be clearly seen that further theoretical and experimental studies are demanded to reveal the exact physical origin. It is our view that such investigations are preferred to be performed from the fundamental single-particle or single-plasmon level via direct characterizations of temperature rise and chemical reaction of the plasmonic structures using a variety of tools such as scanning thermal microscopy [306], [307], [308], single particle spectroscopy [309], and single-molecule chemical reaction imaging [310].

### 6.3 Potential future directions

**Figure 13:**
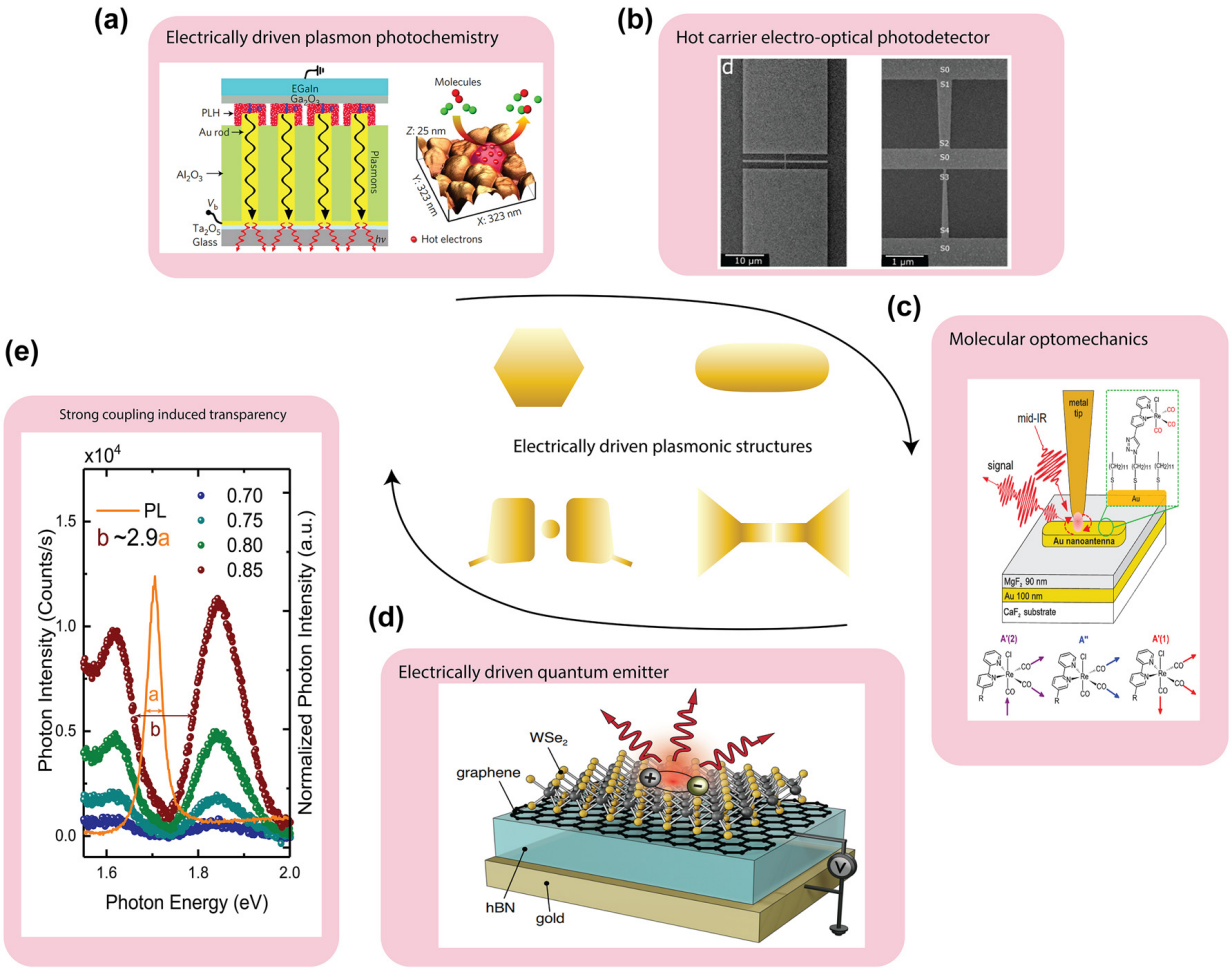
Prospective research topics based on electrically driven plasmonic processes. (a) Electrically driven plasmonic nanorod used as reactive tunnel junction [302]. Reproduced with permission from the Springer Nature Group. (b) Plasmonic-active asymmetric gold nanostructures for photodetectors [271]. Reproduced with permission from the American Chemical Society. (c) Antenna-coupled vibrational spectroscopy based on STM [230]. Reproduced with permission from the National Academy of Science. (d) Hybrid plasmon-exciton coupling system as a quantum emitter [260]. (e) A coupled single molecular junction based on STM shows transparency in the light emission spectra [246]. Reproduced with permission from the Springer Nature Group.

The study of plasmon-induced hot carriers and electrically driven plasmonic processes has emerged over the past decade as one of the major frontiers in nanophotonics and plasmonics. Plasmonic nanostructures made of tunneling nanojunctions and ultrathin nanogaps with only atomic and molecular sizes are not only of fundamental interest to reveal the limits of light–matter interaction at the extreme scale, but also of importance for applications in photonic devices, optoelectronics, and quantum technology, as summarized in the cartoons in Figure 13. The capability of these structures to allow simultaneous control using both optical and electrical methods, which is not possible for conventional nanoparticle based plasmonic structures, has great potential to bridge the ultraminiaturized device footprint of plasmonic circuitry with external photonic networks using well-established on-chip electronic means. Biased plasmonic tunnel junctions serve as an important platform in this regard as it can electrically excite plasmonic excitations and directly read out plasmon-induced hot carrier transport information. However, based on our above discussions, neither theoretical nor experimental studies on these fundamental studies and the applications have reached a mature level. Here, we discuss some potential research directions and challenges.

While our current theoretical framework of plasmon-induced hot carriers has provided insightful tools and basic physical pictures to understand many experimental results in different plasmonic structures, models with quantitative predictive power remain to be developed. As intrinsically complicated open quantum systems, plasmonic and hot carrier processes are typically contributed from multiple factors that are interrelated. Further, the energy transport, conversion, and dissipation in a plasmonic nanostructure often span many spatial and temporal scales, which are difficult to model and obtain robust predictions. There is great interest in addressing the ongoing debate arising due to our limited capability to quantify hot carriers in many plasmonic applications and distinguish the role of hot carriers from other contributing factors.

Experimentally, much progress has been made in our understanding of optically driven hot carriers. However, the study of electrically driven hot carrier generation and relaxation has been initiated comparatively recently. Some pronounced open questions included: what are the relative contributions of multielectron coherent processes and hot carrier recombination in pumping above-threshold photons? Can we distinguish hot carrier and electronic Raman physics [311], [312] in electrically and optically driven plasmonic processes? Can we control quantum plasmonic modes [313] using electronic means? Advanced experimental techniques such as single molecule scanning probe microscopy [119], [314], [315] could serve a very important role to answer these questions by imaging the distribution of hot carriers and tracking the hot carrier dynamics when coupled with ultrafast spectroscopy techniques.

On accessing the strong and ultra-strong regime in plasmonic systems, molecular scale plasmonic nanostructures possesses some advantages over conventional larger scale structures; however, experimental studies in this regime based on optically driven plasmonics have been quite challenging, which impedes potential applications. In this regard, exploring hot carrier mediated plexciton strong coupling in the electrically driven regime could be of great interest. One challenge that needs to be overcome is the typically weak electroluminescence from these hybrid strong coupling systems. Discovering an electrically driven hybrid plexcitonic emission with ultra-high brightness holds significant promise for future endeavors [316].

The application of plasmon-induced hot carriers and electrically driven plasmonics in quantum optics could become a fruitful area. From a theoretical perspective, it is proposed that the intricate plasmonic spectrum of the nanocavity presents avenues for generating nonclassical light through mechanisms that can be more efficient than the resonant interaction between the emitter’s natural transition and the optical mode [317], [318], [319]. It has been predicted that, not only is the photon blockade effect discernible, but also photon antibunching stemming from processes of destructive interference, known as unconventional antibunching, is evident. Furthermore, photon bunching can also occur at particular frequencies for plexciton anti-crossing when the scattered intensity is at minimum, demonstrating the tuning capability for the photon statistics of the hybrid plexcitonic system.
